# Genetic interaction motif finding by expectation maximization – a novel statistical model for inferring gene modules from synthetic lethality

**DOI:** 10.1186/1471-2105-6-288

**Published:** 2005-12-06

**Authors:** Yan Qi, Ping Ye, Joel S Bader

**Affiliations:** 1 Biomedical Engineering Department, Johns Hopkins University, North Charles Street, Baltimore, MD, 21218, USA; 2 High-Throughput Biology Center, Johns Hopkins School of Medicine, 733 North Broadway, Baltimore, MD 21205, USA

## Abstract

**Background:**

Synthetic lethality experiments identify pairs of genes with complementary function. More direct functional associations (for example greater probability of membership in a single protein complex) may be inferred between genes that share synthetic lethal interaction partners than genes that are directly synthetic lethal. Probabilistic algorithms that identify gene modules based on motif discovery are highly appropriate for the analysis of synthetic lethal genetic interaction data and have great potential in integrative analysis of heterogeneous datasets.

**Results:**

We have developed Genetic Interaction Motif Finding (GIMF), an algorithm for unsupervised motif discovery from synthetic lethal interaction data. Interaction motifs are characterized by position weight matrices and optimized through expectation maximization. Given a seed gene, GIMF performs a nonlinear transform on the input genetic interaction data and automatically assigns genes to the motif or non-motif category. We demonstrate the capacity to extract known and novel pathways for Saccharomyces cerevisiae (budding yeast). Annotations suggested for several uncharacterized genes are supported by recent experimental evidence. GIMF is efficient in computation, requires no training and automatically down-weights promiscuous genes with high degrees.

**Conclusion:**

GIMF effectively identifies pathways from synthetic lethality data with several unique features. It is mostly suitable for building gene modules around seed genes. Optimal choice of one single model parameter allows construction of gene networks with different levels of confidence. The impact of hub genes the generic probabilistic framework of GIMF may be used to group other types of biological entities such as proteins based on stochastic motifs. Analysis of the strongest motifs discovered by the algorithm indicates that synthetic lethal interactions are depleted between genes within a motif, suggesting that synthetic lethality occurs between-pathway rather than within-pathway.

## Background

Much recent research efforts have been devoted to studying gene functions in the context of highly dynamic and modular cellular networks [1-4]. Valuable information about a gene's function can be obtained from its interaction with other genes [5]. Apart from the traditional hierarchical way of gene function annotation, functional genomics takes a bottom-up approach to assemble gene interaction networks based on all pair-wise gene interactions detected. From such genetic interaction maps, Functional modules representing various biological pathways and processes can then be extracted by computational approaches. Those modules naturally suggest novel gene functions in the relevant biological processes [6]. The interactions between genes are of course highly dynamic spatially and temporally. However, one of the most intuitive yet fundamental questions about genetic interactions is whether the normal functioning of two genes depends on each other. Synthetic lethality identifies genes that complement each other's function: two genes are synthetic lethal if either single mutant is viable, but the double mutant combination is lethal. High-throughput experiments such as synthetic genetic array (SGA) [7] and synthetic lethality analyzed by microarray (SLAM) [8,9] have been done for genome-wide synthetic lethality analysis on *Saccharomyces cerevisiae*, where a single mutant (query gene) is introduced into the complete pool of viable yeast single-deletion (library gene) strains. Synthetic lethality data obtained through SGA, SLAM or RNA interference has shed much new light on essential biological pathways and the function assignment for many previously uncharacterized genes for the model organisms yeast and C. elegans [10,11]. Hierarchical clustering of the SGA dataset suggest that two synthetic lethal genes are likely to (i) reside in two redundant parallel pathways or (ii) complement each other's function in two branches of one essential pathway [12]. Computational methods integrating physical protein interactions and other genomic features seem to suggest that significantly more synthetic lethal interactions occur between parallel pathways [13,14]. Given the incomplete and error-prone synthetic lethal interaction map, it is highly desirable to investigate methods that extract biologically relevant information probabilistically, which accommodates network properties such as degree distribution and confidence of the links. Along this line, we have developed in this study a probabilistic model for characterizing synthetic lethal interaction motifs and an algorithm that automatically groups genes sharing similar motifs into pathways. When applied to the SGA dataset, our method automatically uncovers known and novel gene modules that correlate favourably with Gene Ontology (GO) annotations.

## Results

### Data sources

Genetic interaction data is obtained from SGA analysis in yeast [12]. The original query gene set includes 126 non-essential genes and 6 essential genes, tested against a library of all non-essential gene deletions. Interpretation of synthetic lethality involving essential genes is problematic since the intermediate (viable) phenotypes exhibited by conditional alleles of essential genes may include loss of function, unregulated function, and gain of function aspects. Thus we focus on synthetic lethal interactions between null alleles of non-essential genes, which by definition result from solely loss of function mutations. Ignoring library genes that have no interaction with any of the 126 query genes, our dataset consists of 126 query genes linked to 982 library genes by 4287 interactions. Both the query and the library sets contain hubs with high interaction counts (Supp. Figs. S3, S4, and S5).

Yeast protein complex data were obtained from two high-throughput studies, TAP and HMS-PCI [15,16]. Protein complexes that contained two or more non-essential proteins were used (353 complexes from TAP and 427 complexes from HMS-PCI).

### Computational method

**Figure 1:**
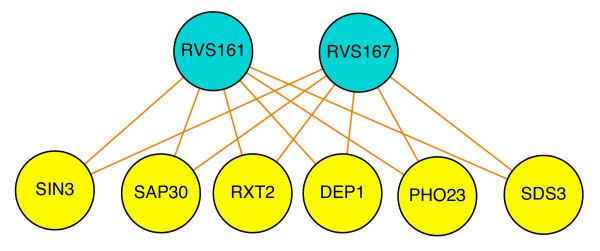
**Synthetic lethal interactions between complementary pathways**. Proteins in the RVS161 complex (RVS161, RVS167) have enriched synthetic lethal interactions (orange lines) with proteins in the RPD3 complex. The RVS161 complex and RPD3 complex are associated with endocytosis/viability following starvation and telomere silencing, respectively.

The Expectation maximization (EM) algorithm has been widely used to detect motifs in biopolymer sequences, where a position weight matrix representing a recurring pattern (such as DNA binding sites or promoter regions) in multiple unaligned sequences is built iteratively by maximum likelihood scoring [17-20]. Such probabilistic approach is most suitable for the detection of patterns with a stochastic nature, which we have little prior knowledge of. In this study, we have developed an algorithm for finding genes in the same pathway, which we shall refer to as Genetic Interaction Motif Finding by expectation maximization (GIMF). Note the difference between motif here defined by genetic interaction pattern and the network topological motifs [21]. The model is developed under the hypothesis that genes within the same pathway exhibit a similar pattern of synthetic lethal interactions where a subset of common interaction partners are genes in complementary pathways [12-14]. For example, RVS161 and RVS167 are two queries that belong to the RVS161 complex. Enhanced synthetic lethal interactions with members of the RPD3 complex have been observed (Fig. 1). The RVS161 complex proteins are AR adaptor proteins involved in actin regulation, endocytosis and viability following starvation or osmotic stress. The RPD3 histone deacetylase complex is involved in silencing at telomeres. In particular, DEP1, a member of the RPD3 complex is a transcriptional modulator of phospholipids biosynthesis and also maintains mating efficiency and sporulation. Thus it is reasonable to infer that these two protein complexes are functionally complementary during endocytosis and mating or sporulation after starvation when the biological processes of the two complexes are tightly coupled.

In our analysis, we focus on finding motifs from the synthetic lethal interaction patterns of query genes. Let *X*_*i*_ = [*X*_*i*1 _... *X*_*iN*_] denote the interaction partner list for query gene *i*, where *X*_*ij *_= 1 if *i *interacts with library gene *j *and *X*_*ij *_= 0 otherwise. Thus the entire data set is *X*_*i*_, *i *= 1,2,...,Q. The total numbers of query is Q = 126 and the total number of the library genes that interact with at least one query gene is *N *= 982. We initiate a search with a query gene s and aim to find all other genes in the same pathway as the seed gene s. We do this by iteratively constructing a motif for the group and hence identifying motif members.

Mathematically, we divide the query gene set into two sets, a motif set *A *={*A*_i_}, *i *= 1,2,...a_*M*_, initialized to contain just the seed gene, and a non-motif set *B *= {*B*_i_}, *i=a*_*M*_+1, a_*M*_+2, L, a_*M*_+b_*M*_, containing the remaining genes. The number of query genes in the motif and non-motif sets are a_*M *_and b_*M*_, respectively, with a_*M*_+b_*M*_=Q. We assume that genes in the motif set and those in the non-motif set have different probabilities of interacting with a library gene *j*, which are denoted by *p*_*aj *_and *p*_*bj*_, respectively. As will be explained in DISCUSSION, this allows existence of hub library genes explicitly. The probability that query *i *belongs to the motif set is denoted by *z*_*i*_. The parameters *p*_*aj*_, *p*_*bj *_and *z*_*i*_, where *j *= 1,2...,*N *and *j *= 1,2...,Q, are estimated iteratively.

The expectation maximization (EM) algorithm has been used for maximum likelihood estimation with missing information. In our scenario, given a seed gene, missing information is represented by the correct partition of the entire gene pool into a motif set *A *and a non-motif set *B *starting from an initial motif estimate provided by the seed. The likelihood function, i.e. the conditional probability of observing measured data given the partition, is

L=P(X|A,B)=∏i=1aM∏j=1N[Xijpaj+(1−Xij)(1−paj)]×∏i=aM+1aM+bM∏j=1N[Xijpbj+(1−Xij)(1−pbj)].     (1)
 MathType@MTEF@5@5@+=feaafiart1ev1aaatCvAUfKttLearuWrP9MDH5MBPbIqV92AaeXatLxBI9gBaebbnrfifHhDYfgasaacH8akY=wiFfYdH8Gipec8Eeeu0xXdbba9frFj0=OqFfea0dXdd9vqai=hGuQ8kuc9pgc9s8qqaq=dirpe0xb9q8qiLsFr0=vr0=vr0dc8meaabaqaciGacaGaaeqabaqabeGadaaakqaaeeqaaiabdYeamjabg2da9iabdcfaqjabcIcaOiabdIfayjabcYha8jabdgeabjabcYcaSiabdkeacjabcMcaPaqaaiabg2da9maarahabaWaaebCaeaadaWadaqaaiabdIfaynaaBaaaleaacqWGPbqAcqWGQbGAaeqaaOGaemiCaa3aaSbaaSqaaiabdggaHjabdQgaQbqabaGccqGHRaWkdaqadaqaaiabigdaXiabgkHiTiabdIfaynaaBaaaleaacqWGPbqAcqWGQbGAaeqaaaGccaGLOaGaayzkaaWaaeWaaeaacqaIXaqmcqGHsislcqWGWbaCdaWgaaWcbaGaemyyaeMaemOAaOgabeaaaOGaayjkaiaawMcaaaGaay5waiaaw2faaaWcbaGaemOAaOMaeyypa0JaeGymaedabaGaemOta4eaniabg+GivdGccqGHxdaTaSqaaiabdMgaPjabg2da9iabigdaXaqaaiabdggaHnaaBaaameaacqWGnbqtaeqaaaqdcqGHpis1aOWaaebCaeaadaqeWbqaamaadmaabaGaemiwaG1aaSbaaSqaaiabdMgaPjabdQgaQbqabaGccqWGWbaCdaWgaaWcbaGaemOyaiMaemOAaOgabeaakiabgUcaRmaabmaabaGaeGymaeJaeyOeI0IaemiwaG1aaSbaaSqaaiabdMgaPjabdQgaQbqabaaakiaawIcacaGLPaaadaqadaqaaiabigdaXiabgkHiTiabdchaWnaaBaaaleaacqWGIbGycqWGQbGAaeqaaaGccaGLOaGaayzkaaaacaGLBbGaayzxaaaaleaacqWGQbGAcqGH9aqpcqaIXaqmaeaacqWGobGta0Gaey4dIunaaSqaaiabdMgaPjabg2da9iabdggaHnaaBaaameaacqWGnbqtaeqaaSGaey4kaSIaeGymaedabaGaemyyae2aaSbaaWqaaiabd2eanbqabaWccqGHRaWkcqWGIbGydaWgaaadbaGaemyta0eabeaaa0Gaey4dIunakiabb6caUiaaxMaacaWLjaWaaeWaaeaacqqGXaqmaiaawIcacaGLPaaaaaaa@98D9@

Thus the log likelihood function is

log⁡L=∑j=1N∑i=1aM[Xijlog⁡paj+(1−Xij)log⁡(1−paj)]+∑j=1N∑i=aM+1aM+bM[Xijlog⁡pbj+(1−Xij)log⁡(1−pbj)]=aM∑j=1N[fajlog⁡paj+(1−faj)log⁡(1−paj)]+bM∑j=1N[fbjlog⁡pbj+(1−fbj)log⁡(1−pbj)]     (2)
 MathType@MTEF@5@5@+=feaafiart1ev1aaatCvAUfKttLearuWrP9MDH5MBPbIqV92AaeXatLxBI9gBaebbnrfifHhDYfgasaacH8akY=wiFfYdH8Gipec8Eeeu0xXdbba9frFj0=OqFfea0dXdd9vqai=hGuQ8kuc9pgc9s8qqaq=dirpe0xb9q8qiLsFr0=vr0=vr0dc8meaabaqaciGacaGaaeqabaqabeGadaaakeaafaqaceWabaaaeaqabeaacyGGSbaBcqGGVbWBcqGGNbWzcqWGmbatcqGH9aqpdaaeWbqaamaaqahabaWaamWaaeaacqWGybawdaWgaaWcbaGaemyAaKMaemOAaOgabeaakiGbcYgaSjabc+gaVjabcEgaNjabdchaWnaaBaaaleaacqWGHbqycqWGQbGAaeqaaOGaey4kaSYaaeWaaeaacqaIXaqmcqGHsislcqWGybawdaWgaaWcbaGaemyAaKMaemOAaOgabeaaaOGaayjkaiaawMcaaiGbcYgaSjabc+gaVjabcEgaNnaabmaabaGaeGymaeJaeyOeI0IaemiCaa3aaSbaaSqaaiabdggaHjabdQgaQbqabaaakiaawIcacaGLPaaaaiaawUfacaGLDbaaaSqaaiabdMgaPjabg2da9iabigdaXaqaaiabdggaHnaaBaaameaacqWGnbqtaeqaaaqdcqGHris5aaWcbaGaemOAaOMaeyypa0JaeGymaedabaGaemOta4eaniabggHiLdaakeaacWaGaItaaaGHRaWkdGaGaItaaaaeWbqaiaiG4eaaamacaciobaaaqahabGaGaItaaaWaiaiG4eaaamWaaeacaciobaaacWaGaItaaaWGybawdGaGaItaaaWgaaWcbGaGaItaaaGamaiG4eaaamyAaKMamaiG4eaaamOAaOgabKaGaItaaaaakiGdaciobaaacYgaSjadaciobaaac+gaVjadaciobaaacEgaNjadaciobaaadchaWnacaciobaaaBaaaleacaciobaaacWaGaItaaaWGIbGycWaGaItaaaWGQbGAaeqcaciobaaaaOGamaiG4eaaay4kaSYaiaiG4eaaaeWaaeacaciobaaacWaGaItaaaaIXaqmcWaGaItaaaGHsislcWaGaItaaaWGybawdGaGaItaaaWgaaWcbGaGaItaaaGamaiG4eaaamyAaKMamaiG4eaaamOAaOgabKaGaItaaaaaaOGaiaiG4eaaayjkaiacaciobaaawMcaaiGdaciobaaacYgaSjadaciobaaac+gaVjadaciobaaacEgaNnacaciobaaabmaabGaGaItaaaGamaiG4eaaaGymaeJamaiG4eaaayOeI0IamaiG4eaaamiCaa3aiaiG4eaaaSbaaSqaiaiG4eaaaiadaciobaaadkgaIjadaciobaaadQgaQbqajaiG4eaaaaaakiacaciobaaawIcacGaGaItaaaGLPaaaaiacaciobaaawUfacGaGaItaaaGLDbaaaSqaiaiG4eaaaiadaciobaaadMgaPjadaciobaaag2da9iadaciobaaadggaHnacaciobaaaBaaameacaciobaaacWaGaItaaaWGnbqtaeqcaciobaaaaSGamaiG4eaaay4kaSIamaiG4eaaaGymaedabGaGaItaaaGamaiG4eaaamyyae2aiaiG4eaaaSbaaWqaiaiG4eaaaiadaciobaaad2eanbqajaiG4eaaaaWccWaGaItaaaGHRaWkcWaGaItaaaWGIbGydGaGaItaaaWgaaadbGaGaItaaaGamaiG4eaaamyta0eabKaGaItaaaaaa0GamaiG4eaaayyeIuoaaSqaiaiG4eaaaiadaciobaaadQgaQjadaciobaaag2da9iadaciobaaaigdaXaqaiaiG4eaaaiadaciobaaad6eaobqdcWaGaItaaaGHris5aaaakeaacqGH9aqpcqWGHbqydaWgaaWcbaGaemyta0eabeaakmaaqahabaWaamWaaeaacqWGMbGzdaWgaaWcbaGaemyyaeMaemOAaOgabeaakiGbcYgaSjabc+gaVjabcEgaNjabdchaWnaaBaaaleaacqWGHbqycqWGQbGAaeqaaOGaey4kaSIaeiikaGIaeGymaeJaeyOeI0IaemOzay2aaSbaaSqaaiabdggaHjabdQgaQbqabaGccqGGPaqkcyGGSbaBcqGGVbWBcqGGNbWzcqGGOaakcqaIXaqmcqGHsislcqWGWbaCdaWgaaWcbaGaemyyaeMaemOAaOgabeaakiabcMcaPaGaay5waiaaw2faaaWcbaGaemOAaOMaeyypa0JaeGymaedabaGaemOta4eaniabggHiLdaakeaacqGHRaWkcqWGIbGydaWgaaWcbaGaemyta0eabeaakmaaqahabaWaamWaaeaacqWGMbGzdaWgaaWcbaGaemOyaiMaemOAaOgabeaakiGbcYgaSjabc+gaVjabcEgaNjabdchaWnaaBaaaleaacqWGIbGycqWGQbGAaeqaaOGaey4kaSIaeiikaGIaeGymaeJaeyOeI0IaemOzay2aaSbaaSqaaiabdkgaIjabdQgaQbqabaGccqGGPaqkcyGGSbaBcqGGVbWBcqGGNbWzcqGGOaakcqaIXaqmcqGHsislcqWGWbaCdaWgaaWcbaGaemOyaiMaemOAaOgabeaakiabcMcaPaGaay5waiaaw2faaaWcbaGaemOAaOMaeyypa0JaeGymaedabaGaemOta4eaniabggHiLdaaaOGaaCzcaiaaxMaadGaGacaaK3FadaqaiaiGaaa59ladaciaaqE=ikdaYaGaiaiGaaa59BjkaiacaciaaqE=wMcaaaaa@727A@

where faj=1aM∑i=1aMXij=najaM
 MathType@MTEF@5@5@+=feaafiart1ev1aaatCvAUfKttLearuWrP9MDH5MBPbIqV92AaeXatLxBI9gBaebbnrfifHhDYfgasaacH8akY=wiFfYdH8Gipec8Eeeu0xXdbba9frFj0=OqFfea0dXdd9vqai=hGuQ8kuc9pgc9s8qqaq=dirpe0xb9q8qiLsFr0=vr0=vr0dc8meaabaqaciGacaGaaeqabaqabeGadaaakeaacqWGMbGzdaWgaaWcbaGaemyyaeMaemOAaOgabeaakiabg2da9maalaaabaGaeGymaedabaGaemyyae2aaSbaaSqaaiabd2eanbqabaaaaOWaaabCaeaacqWGybawdaWgaaWcbaGaemyAaKMaemOAaOgabeaaaeaacqWGPbqAcqGH9aqpcqaIXaqmaeaacqWGHbqydaWgaaadbaGaemyta0eabeaaa0GaeyyeIuoakiabg2da9maalaaabaGaemOBa42aaSbaaSqaaiabdggaHjabdQgaQbqabaaakeaacqWGHbqydaWgaaWcbaGaemyta0eabeaaaaaaaa@49C8@ and fbj=1bM∑i=aM+1aM+bMXij=nbjbM
 MathType@MTEF@5@5@+=feaafiart1ev1aaatCvAUfKttLearuWrP9MDH5MBPbIqV92AaeXatLxBI9gBaebbnrfifHhDYfgasaacH8akY=wiFfYdH8Gipec8Eeeu0xXdbba9frFj0=OqFfea0dXdd9vqai=hGuQ8kuc9pgc9s8qqaq=dirpe0xb9q8qiLsFr0=vr0=vr0dc8meaabaqaciGacaGaaeqabaqabeGadaaakeaacqWGMbGzdaWgaaWcbaGaemOyaiMaemOAaOgabeaakiabg2da9maalaaabaGaeGymaedabaGaemOyai2aaSbaaSqaaiabd2eanbqabaaaaOWaaabCaeaacqWGybawdaWgaaWcbaGaemyAaKMaemOAaOgabeaaaeaacqWGPbqAcqGH9aqpcqWGHbqydaWgaaadbaGaemyta0eabeaaliabgUcaRiabigdaXaqaaiabdggaHnaaBaaameaacqWGnbqtaeqaaSGaey4kaSIaemOyai2aaSbaaWqaaiabd2eanbqabaaaniabggHiLdGccqGH9aqpdaWcaaqaaiabd6gaUnaaBaaaleaacqWGIbGycqWGQbGAaeqaaaGcbaGaemOyai2aaSbaaSqaaiabd2eanbqabaaaaaaa@50E2@ are the observed frequencies of the interaction between the library gene *j *and query genes in motif set and non-motif set, respectively. Maximizing the log likelihood function, we obtain the maximum likelihood estimates for *p*_*aj *_and *p*_*bj*_, which are simply the sample frequencies, i.e. p^aj=faj
 MathType@MTEF@5@5@+=feaafiart1ev1aaatCvAUfKttLearuWrP9MDH5MBPbIqV92AaeXatLxBI9gBaebbnrfifHhDYfgasaacH8akY=wiFfYdH8Gipec8Eeeu0xXdbba9frFj0=OqFfea0dXdd9vqai=hGuQ8kuc9pgc9s8qqaq=dirpe0xb9q8qiLsFr0=vr0=vr0dc8meaabaqaciGacaGaaeqabaqabeGadaaakeaacuWGWbaCgaqcamaaBaaaleaacqWGHbqycqWGQbGAaeqaaOGaeyypa0JaemOzay2aaSbaaSqaaiabdggaHjabdQgaQbqabaaaaa@3634@ and p^bj=fbj
 MathType@MTEF@5@5@+=feaafiart1ev1aaatCvAUfKttLearuWrP9MDH5MBPbIqV92AaeXatLxBI9gBaebbnrfifHhDYfgasaacH8akY=wiFfYdH8Gipec8Eeeu0xXdbba9frFj0=OqFfea0dXdd9vqai=hGuQ8kuc9pgc9s8qqaq=dirpe0xb9q8qiLsFr0=vr0=vr0dc8meaabaqaciGacaGaaeqabaqabeGadaaakeaacuWGWbaCgaqcamaaBaaaleaacqWGIbGycqWGQbGAaeqaaOGaeyypa0JaemOzay2aaSbaaSqaaiabdkgaIjabdQgaQbqabaaaaa@3638@ (unless otherwise stated, an overlying hat denotes the maximum likelihood estimate of a parameter). These estimates cannot be obtained when the partition information is missing. The EM algorithm starts with an initial guess of the solution and iteratively updates the model parameters with expected information obtained by maximum likelihood estimation in each step. More specifically, each iteration comprises two steps, an expectation (E) step and a maximization (M) step.

Let us assume that q iterations have been completed. At the start of the E-step of iteration q+1, the estimates for the model parameters from the M step of the previous iteration, p^a(q)
 MathType@MTEF@5@5@+=feaafiart1ev1aaatCvAUfKttLearuWrP9MDH5MBPbIqV92AaeXatLxBI9gBaebbnrfifHhDYfgasaacH8akY=wiFfYdH8Gipec8Eeeu0xXdbba9frFj0=OqFfea0dXdd9vqai=hGuQ8kuc9pgc9s8qqaq=dirpe0xb9q8qiLsFr0=vr0=vr0dc8meaabaqaciGacaGaaeqabaqabeGadaaakeaacuWGWbaCgaqcamaaDaaaleaacqWGHbqyaeaacqGGOaakcqWGXbqCcqGGPaqkaaaaaa@32BC@, p^b(q)
 MathType@MTEF@5@5@+=feaafiart1ev1aaatCvAUfKttLearuWrP9MDH5MBPbIqV92AaeXatLxBI9gBaebbnrfifHhDYfgasaacH8akY=wiFfYdH8Gipec8Eeeu0xXdbba9frFj0=OqFfea0dXdd9vqai=hGuQ8kuc9pgc9s8qqaq=dirpe0xb9q8qiLsFr0=vr0=vr0dc8meaabaqaciGacaGaaeqabaqabeGadaaakeaacuWGWbaCgaqcamaaDaaaleaacqWGIbGyaeaacqGGOaakcqWGXbqCcqGGPaqkaaaaaa@32BE@ and z^i(q)
 MathType@MTEF@5@5@+=feaafiart1ev1aaatCvAUfKttLearuWrP9MDH5MBPbIqV92AaeXatLxBI9gBaebbnrfifHhDYfgasaacH8akY=wiFfYdH8Gipec8Eeeu0xXdbba9frFj0=OqFfea0dXdd9vqai=hGuQ8kuc9pgc9s8qqaq=dirpe0xb9q8qiLsFr0=vr0=vr0dc8meaabaqaciGacaGaaeqabaqabeGadaaakeaacuWG6bGEgaqcamaaDaaaleaacqWGPbqAaeaacqGGOaakcqWGXbqCcqGGPaqkaaaaaa@32E0@, are available. Let Y_*i *_be a motif indicator, i.e. Y_*i *_= 1 if gene *i *belongs to the motif set and Y_*i *_= 0 otherwise. Then the conditional probability of observing X⇀i
 MathType@MTEF@5@5@+=feaafiart1ev1aaatCvAUfKttLearuWrP9MDH5MBPbIqV92AaeXatLxBI9gBaebbnrfifHhDYfgasaacH8akY=wiFfYdH8Gipec8Eeeu0xXdbba9frFj0=OqFfea0dXdd9vqai=hGuQ8kuc9pgc9s8qqaq=dirpe0xb9q8qiLsFr0=vr0=vr0dc8meaabaqaciGacaGaaeqabaqabeGadaaakeaacuWGybawgaGdamaaBaaaleaacqWGPbqAaeqaaaaa@2F83@, given that gene *i *belongs to the motif set and p^a(q)
 MathType@MTEF@5@5@+=feaafiart1ev1aaatCvAUfKttLearuWrP9MDH5MBPbIqV92AaeXatLxBI9gBaebbnrfifHhDYfgasaacH8akY=wiFfYdH8Gipec8Eeeu0xXdbba9frFj0=OqFfea0dXdd9vqai=hGuQ8kuc9pgc9s8qqaq=dirpe0xb9q8qiLsFr0=vr0=vr0dc8meaabaqaciGacaGaaeqabaqabeGadaaakeaacuWGWbaCgaqcamaaDaaaleaacqWGHbqyaeaacqGGOaakcqWGXbqCcqGGPaqkaaaaaa@32BC@, is

P(Xi|Yi=1,p^a(q))=∏j=1N[p^aj(q)Xij+(1−p^aj(q))(1−Xij)].     (3)
 MathType@MTEF@5@5@+=feaafiart1ev1aaatCvAUfKttLearuWrP9MDH5MBPbIqV92AaeXatLxBI9gBaebbnrfifHhDYfgasaacH8akY=wiFfYdH8Gipec8Eeeu0xXdbba9frFj0=OqFfea0dXdd9vqai=hGuQ8kuc9pgc9s8qqaq=dirpe0xb9q8qiLsFr0=vr0=vr0dc8meaabaqaciGacaGaaeqabaqabeGadaaakeaacqqGqbaucqGGOaakcqWGybawdaWgaaWcbaGaemyAaKgabeaakiabcYha8jabdMfaznaaBaaaleaacqWGPbqAaeqaaOGaeyypa0JaeGymaeJaeiilaWIafmiCaaNbaKaadaqhaaWcbaGaemyyaegabaGaeiikaGIaemyCaeNaeiykaKcaaOGaeiykaKIaeyypa0ZaaybCaeqaleaacqWGQbGAcqGH9aqpcqaIXaqmaeaacqWGobGta0qaaiabg+GivdaakmaadmaabaGafmiCaaNbaKaadaqhaaWcbaGaemyyaeMaemOAaOgabaGaeiikaGIaemyCaeNaeiykaKcaaOGaemiwaG1aaSbaaSqaaiabdMgaPjabdQgaQbqabaGccqGHRaWkcqGGOaakcqaIXaqmcqGHsislcuWGWbaCgaqcamaaDaaaleaacqWGHbqycqWGQbGAaeaacqGGOaakcqWGXbqCcqGGPaqkaaGccqGGPaqkcqGGOaakcqaIXaqmcqGHsislcqWGybawdaWgaaWcbaGaemyAaKMaemOAaOgabeaakiabcMcaPaGaay5waiaaw2faaiabc6caUiaaxMaacaWLjaWaaeWaaeaacqaIZaWmaiaawIcacaGLPaaaaaa@6D1A@

Similarly the conditional probability of observing X⇀i
 MathType@MTEF@5@5@+=feaafiart1ev1aaatCvAUfKttLearuWrP9MDH5MBPbIqV92AaeXatLxBI9gBaebbnrfifHhDYfgasaacH8akY=wiFfYdH8Gipec8Eeeu0xXdbba9frFj0=OqFfea0dXdd9vqai=hGuQ8kuc9pgc9s8qqaq=dirpe0xb9q8qiLsFr0=vr0=vr0dc8meaabaqaciGacaGaaeqabaqabeGadaaakeaacuWGybawgaGdamaaBaaaleaacqWGPbqAaeqaaaaa@2F83@ given that gene *i *belongs to the non-motif set and p^b(q)
 MathType@MTEF@5@5@+=feaafiart1ev1aaatCvAUfKttLearuWrP9MDH5MBPbIqV92AaeXatLxBI9gBaebbnrfifHhDYfgasaacH8akY=wiFfYdH8Gipec8Eeeu0xXdbba9frFj0=OqFfea0dXdd9vqai=hGuQ8kuc9pgc9s8qqaq=dirpe0xb9q8qiLsFr0=vr0=vr0dc8meaabaqaciGacaGaaeqabaqabeGadaaakeaacuWGWbaCgaqcamaaDaaaleaacqWGIbGyaeaacqGGOaakcqWGXbqCcqGGPaqkaaaaaa@32BE@, is

P(Xi|Yi=0,p^b(q))=∏j=1N[p^bj(q)Xij+(1−p^bj(q))(1−Xij)].     (4)
 MathType@MTEF@5@5@+=feaafiart1ev1aaatCvAUfKttLearuWrP9MDH5MBPbIqV92AaeXatLxBI9gBaebbnrfifHhDYfgasaacH8akY=wiFfYdH8Gipec8Eeeu0xXdbba9frFj0=OqFfea0dXdd9vqai=hGuQ8kuc9pgc9s8qqaq=dirpe0xb9q8qiLsFr0=vr0=vr0dc8meaabaqaciGacaGaaeqabaqabeGadaaakeaaieaacqWFqbaucqGGOaakcqWGybawdaWgaaWcbaGaemyAaKgabeaakiabcYha8jabdMfaznaaBaaaleaacqWGPbqAaeqaaOGaeyypa0JaeGimaaJaeiilaWIafmiCaaNbaKaadaqhaaWcbaGaemOyaigabaGaeiikaGIaemyCaeNaeiykaKcaaOGaeiykaKIaeyypa0ZaaybCaeqaleaacqWGQbGAcqGH9aqpcqaIXaqmaeaacqWGobGta0qaaiabg+GivdaakmaadmaabaGafmiCaaNbaKaadaqhaaWcbaGaemOyaiMaemOAaOgabaGaeiikaGIaemyCaeNaeiykaKcaaOGaemiwaG1aaSbaaSqaaiabdMgaPjabdQgaQbqabaGccqGHRaWkcqGGOaakcqaIXaqmcqGHsislcuWGWbaCgaqcamaaDaaaleaacqWGIbGycqWGQbGAaeaacqGGOaakcqWGXbqCcqGGPaqkaaGccqGGPaqkcqGGOaakcqaIXaqmcqGHsislcqWGybawdaWgaaWcbaGaemyAaKMaemOAaOgabeaakiabcMcaPaGaay5waiaaw2faaiabc6caUiaaxMaacaWLjaWaaeWaaeaacqaI0aanaiaawIcacaGLPaaaaaa@6D27@

By Bayes formula, the probability that a gene *i *belongs to the motif set given observed data and current model estimates is,

z^i(q+1)=P(Yi=1|Xi,p^a(q),p^b(q))=P(Xi|Yi=1,p^a(q))P0(Yi=1)P(Xi|Yi=1,p^a(q))P0(Yi=1)+P(Xi|Yi=0,p^b(q))P0(Yi=0),     (5)
 MathType@MTEF@5@5@+=feaafiart1ev1aaatCvAUfKttLearuWrP9MDH5MBPbIqV92AaeXatLxBI9gBaebbnrfifHhDYfgasaacH8akY=wiFfYdH8Gipec8Eeeu0xXdbba9frFj0=OqFfea0dXdd9vqai=hGuQ8kuc9pgc9s8qqaq=dirpe0xb9q8qiLsFr0=vr0=vr0dc8meaabaqaciGacaGaaeqabaqabeGadaaakqaaeeqaaiqbdQha6zaajaWaa0baaSqaaiabdMgaPbqaaiabcIcaOiabdghaXjabgUcaRiabigdaXiabcMcaPaaakiabg2da9iabdcfaqjabcIcaOiabdMfaznaaBaaaleaacqWGPbqAaeqaaOGaeyypa0JaeGymaeJaeiiFaWNaemiwaG1aaSbaaSqaaiabdMgaPbqabaGccqGGSaalcuWGWbaCgaqcamaaDaaaleaacqWGHbqyaeaacqGGOaakcqWGXbqCcqGGPaqkaaGccqGGSaalcuWGWbaCgaqcamaaDaaaleaacqWGIbGyaeaacqGGOaakcqWGXbqCcqGGPaqkaaGccqGGPaqkaeaacqGH9aqpdaWcaaqaaiabdcfaqjabcIcaOiabdIfaynaaBaaaleaacqWGPbqAaeqaaOGaeiiFaWNaemywaK1aaSbaaSqaaiabdMgaPbqabaGccqGH9aqpcqaIXaqmcqGGSaalcuWGWbaCgaqcamaaDaaaleaacqWGHbqyaeaacqGGOaakcqWGXbqCcqGGPaqkaaGccqGGPaqkcqWGqbaudaWgaaWcbaGaeGimaadabeaakiabcIcaOiabdMfaznaaBaaaleaacqWGPbqAaeqaaOGaeyypa0JaeGymaeJaeiykaKcabaGaemiuaaLaeiikaGIaemiwaG1aaSbaaSqaaiabdMgaPbqabaGccqGG8baFcqWGzbqwdaWgaaWcbaGaemyAaKgabeaakiabg2da9iabigdaXiabcYcaSiqbdchaWzaajaWaa0baaSqaaiabdggaHbqaaiabcIcaOiabdghaXjabcMcaPaaakiabcMcaPiabdcfaqnaaBaaaleaacqaIWaamaeqaaOGaeiikaGIaemywaK1aaSbaaSqaaiabdMgaPbqabaGccqGH9aqpcqaIXaqmcqGGPaqkcqGHRaWkcqWGqbaucqGGOaakcqWGybawdaWgaaWcbaGaemyAaKgabeaakiabcYha8jabdMfaznaaBaaaleaacqWGPbqAaeqaaOGaeyypa0JaeGimaaJaeiilaWIafmiCaaNbaKaadaqhaaWcbaGaemOyaigabaGaeiikaGIaemyCaeNaeiykaKcaaOGaeiykaKIaemiuaa1aaSbaaSqaaiabicdaWaqabaGccqGGOaakcqWGzbqwdaWgaaWcbaGaemyAaKgabeaakiabg2da9iabicdaWiabcMcaPaaacqGGSaalcaWLjaGaaCzcamaabmaabaGaeGynaudacaGLOaGaayzkaaaaaaa@A920@

where P0(Yi=1)=a^M(0)/Q
 MathType@MTEF@5@5@+=feaafiart1ev1aaatCvAUfKttLearuWrP9MDH5MBPbIqV92AaeXatLxBI9gBaebbnrfifHhDYfgasaacH8akY=wiFfYdH8Gipec8Eeeu0xXdbba9frFj0=OqFfea0dXdd9vqai=hGuQ8kuc9pgc9s8qqaq=dirpe0xb9q8qiLsFr0=vr0=vr0dc8meaabaqaciGacaGaaeqabaqabeGadaaakeaacqWGqbaudaWgaaWcbaGaeGimaadabeaakiabcIcaOiabdMfaznaaBaaaleaacqWGPbqAaeqaaOGaeyypa0JaeGymaeJaeiykaKIaeyypa0JafmyyaeMbaKaadaqhaaWcbaGaemyta0eabaGaeiikaGIaeGimaaJaeiykaKcaaOGaei4la8Iaemyuaefaaa@3DDB@ is the prior probability that gene *i *belongs to the motif set.

The expected number of interactions with a library gene *j *is the weighted sum of all the query genes' interactions with gene *j*, where X_*ij *_is weighted by zi(q+1)
 MathType@MTEF@5@5@+=feaafiart1ev1aaatCvAUfKttLearuWrP9MDH5MBPbIqV92AaeXatLxBI9gBaebbnrfifHhDYfgasaacH8akY=wiFfYdH8Gipec8Eeeu0xXdbba9frFj0=OqFfea0dXdd9vqai=hGuQ8kuc9pgc9s8qqaq=dirpe0xb9q8qiLsFr0=vr0=vr0dc8meaabaqaciGacaGaaeqabaqabeGadaaakeaacqWG6bGEdaqhaaWcbaGaemyAaKgabaGaeiikaGIaemyCaeNaey4kaSIaeGymaeJaeiykaKcaaaaa@34A2@, *i = 1, 2, L, N*. These expected numbers ε_*aj *_and ε_*bj *_for motif and non-motif query genes for iteration *q *+ 1 are

εaj(q+1)=E(naj|X,paj(q))=∑i=1Qzi(q+1)⋅Xij ;εbj(q+1)=E(nbj|X,pbj(q))=∑i=1Q(1−zi(q+1))⋅Xij .     (6)
 MathType@MTEF@5@5@+=feaafiart1ev1aaatCvAUfKttLearuWrP9MDH5MBPbIqV92AaeXatLxBI9gBaebbnrfifHhDYfgasaacH8akY=wiFfYdH8Gipec8Eeeu0xXdbba9frFj0=OqFfea0dXdd9vqai=hGuQ8kuc9pgc9s8qqaq=dirpe0xb9q8qiLsFr0=vr0=vr0dc8meaabaqaciGacaGaaeqabaqabeGadaaakqaabeqaaGGaaiab=v7aLnaaDaaaleaacqWGHbqycqWGQbGAaeaacqGGOaakcqWGXbqCcqGHRaWkcqaIXaqmcqGGPaqkaaGccqGH9aqpieaacqGFfbqrdaqadaqaaiabd6gaUnaaBaaaleaacqWGHbqycqWGQbGAaeqaaOGaeiiFaWNaemiwaGLaeiilaWIaemiCaa3aa0baaSqaaiabdggaHjabdQgaQbqaaiabcIcaOiabdghaXjabcMcaPaaaaOGaayjkaiaawMcaaiabg2da9maaqahabaGaemOEaO3aa0baaSqaaiabdMgaPbqaaiabcIcaOiabdghaXjabgUcaRiabigdaXiabcMcaPaaakiabgwSixlabdIfaynaaBaaaleaacqWGPbqAcqWGQbGAaeqaaOGaeeiiaacaleaacqWGPbqAcqGH9aqpcqaIXaqmaeaacqWGrbqua0GaeyyeIuoakiabcUda7aqaaiab=v7aLnaaDaaaleaacqWGIbGycqWGQbGAaeaacqGGOaakcqWGXbqCcqGHRaWkcqaIXaqmcqGGPaqkaaGccqGH9aqpcqGFfbqrdaqadaqaaiabd6gaUnaaBaaaleaacqWGIbGycqWGQbGAaeqaaOGaeiiFaWNaemiwaGLaeiilaWIaemiCaa3aa0baaSqaaiabdkgaIjabdQgaQbqaaiabcIcaOiabdghaXjabcMcaPaaaaOGaayjkaiaawMcaaiabg2da9maaqahabaWaaeWaaeaacqaIXaqmcqGHsislcqWG6bGEdaqhaaWcbaGaemyAaKgabaGaeiikaGIaemyCaeNaey4kaSIaeGymaeJaeiykaKcaaaGccaGLOaGaayzkaaGaeyyXICTaemiwaG1aaSbaaSqaaiabdMgaPjabdQgaQbqabaaabaGaemyAaKMaeyypa0JaeGymaedabaGaemyuaefaniabggHiLdGccqqGGaaicqqGUaGlcaWLjaGaaCzcamacaciaaGD=bmaabGaGacaay3VamaiGaaa29hOnaydacGaGacaay3VLOaGaiaiGaaa29Bzkaaaaaaa@AAB5@

In the M step, model parameters are updated with expected numbers,

p^aj(q+1)=εaj(q+1)/a^M(q+1);p^bj(q+1)=εbj(q+1)/b^M(q+1);     (7)
 MathType@MTEF@5@5@+=feaafiart1ev1aaatCvAUfKttLearuWrP9MDH5MBPbIqV92AaeXatLxBI9gBaebbnrfifHhDYfgasaacH8akY=wiFfYdH8Gipec8Eeeu0xXdbba9frFj0=OqFfea0dXdd9vqai=hGuQ8kuc9pgc9s8qqaq=dirpe0xb9q8qiLsFr0=vr0=vr0dc8meaabaqaciGacaGaaeqabaqabeGadaaakqaabeqaaiqbdchaWzaajaWaa0baaSqaaiabdggaHjabdQgaQbqaaiabcIcaOiabdghaXjabgUcaRiabigdaXiabcMcaPaaakiabg2da9GGaaiab=v7aLnaaDaaaleaacqWGHbqycqWGQbGAaeaacqGGOaakcqWGXbqCcqGHRaWkcqaIXaqmcqGGPaqkaaGccqGGVaWlcuWGHbqygaqcamaaDaaaleaacqWGnbqtaeaacqGGOaakcqWGXbqCcqGHRaWkcqaIXaqmcqGGPaqkaaGccqGG7aWoaeaacuWGWbaCgaqcamaaDaaaleaacqWGIbGycqWGQbGAaeaacqGGOaakcqWGXbqCcqGHRaWkcqaIXaqmcqGGPaqkaaGccqGH9aqpcqWF1oqzdaqhaaWcbaGaemOyaiMaemOAaOgabaGaeiikaGIaemyCaeNaey4kaSIaeGymaeJaeiykaKcaaOGaei4la8IafmOyaiMbaKaadaqhaaWcbaGaemyta0eabaGaeiikaGIaemyCaeNaey4kaSIaeGymaeJaeiykaKcaaOGaei4oaSJaaCzcaiaaxMaadGaGacaaC5FadaqaiaiGaaax+ladaciaaWL=iEda3aGaiaiGaaax+BjkaiacaciaaWL=wMcaaaaaaa@7702@

where a^M(q+1)=∑i=1Qzi(q+1),b^M(q+1)=Q−a^M(q+1)
 MathType@MTEF@5@5@+=feaafiart1ev1aaatCvAUfKttLearuWrP9MDH5MBPbIqV92AaeXatLxBI9gBaebbnrfifHhDYfgasaacH8akY=wiFfYdH8Gipec8Eeeu0xXdbba9frFj0=OqFfea0dXdd9vqai=hGuQ8kuc9pgc9s8qqaq=dirpe0xb9q8qiLsFr0=vr0=vr0dc8meaabaqaciGacaGaaeqabaqabeGadaaakeaacuWGHbqygaqcamaaDaaaleaacqWGnbqtaeaacqGGOaakcqWGXbqCcqGHRaWkcqaIXaqmcqGGPaqkaaGccqGH9aqpdaaeWbqaaiabdQha6naaDaaaleaacqWGPbqAaeaacqGGOaakcqWGXbqCcqGHRaWkcqaIXaqmcqGGPaqkaaaabaGaemyAaKMaeyypa0JaeGymaedabaGaemyuaefaniabggHiLdGccqGGSaalcuWGIbGygaqcamaaDaaaleaacqWGnbqtaeaacqGGOaakcqWGXbqCcqGHRaWkcqaIXaqmcqGGPaqkaaGccqGH9aqpcqWGrbqucqGHsislcuWGHbqygaqcamaaDaaaleaacqWGnbqtaeaacqGGOaakcqWGXbqCcqGHRaWkcqaIXaqmcqGGPaqkaaaaaa@5747@.

Convergence of the algorithm is assessed by |*t*^(*q*+1) ^- *t*^(*q*)^| < 10^-4 ^for all of the model parameter estimates p^a
 MathType@MTEF@5@5@+=feaafiart1ev1aaatCvAUfKttLearuWrP9MDH5MBPbIqV92AaeXatLxBI9gBaebbnrfifHhDYfgasaacH8akY=wiFfYdH8Gipec8Eeeu0xXdbba9frFj0=OqFfea0dXdd9vqai=hGuQ8kuc9pgc9s8qqaq=dirpe0xb9q8qiLsFr0=vr0=vr0dc8meaabaqaciGacaGaaeqabaqabeGadaaakeaacuWGWbaCgaqcamaaBaaaleaacqWGHbqyaeqaaaaa@2F9E@, p^b
 MathType@MTEF@5@5@+=feaafiart1ev1aaatCvAUfKttLearuWrP9MDH5MBPbIqV92AaeXatLxBI9gBaebbnrfifHhDYfgasaacH8akY=wiFfYdH8Gipec8Eeeu0xXdbba9frFj0=OqFfea0dXdd9vqai=hGuQ8kuc9pgc9s8qqaq=dirpe0xb9q8qiLsFr0=vr0=vr0dc8meaabaqaciGacaGaaeqabaqabeGadaaakeaacuWGWbaCgaqcamaaBaaaleaacqWGIbGyaeqaaaaa@2FA0@ and z^
 MathType@MTEF@5@5@+=feaafiart1ev1aaatCvAUfKttLearuWrP9MDH5MBPbIqV92AaeXatLxBI9gBaebbnrfifHhDYfgasaacH8akY=wiFfYdH8Gipec8Eeeu0xXdbba9frFj0=OqFfea0dXdd9vqai=hGuQ8kuc9pgc9s8qqaq=dirpe0xb9q8qiLsFr0=vr0=vr0dc8meaabaqaciGacaGaaeqabaqabeGadaaakeaacuWG6bGEgaqcaaaa@2E3B@.

Given a seed gene *s*, the model parameters are initialized as follows:

p^bj(0)=Q−1∑i=1QXij     (8)
 MathType@MTEF@5@5@+=feaafiart1ev1aaatCvAUfKttLearuWrP9MDH5MBPbIqV92AaeXatLxBI9gBaebbnrfifHhDYfgasaacH8akY=wiFfYdH8Gipec8Eeeu0xXdbba9frFj0=OqFfea0dXdd9vqai=hGuQ8kuc9pgc9s8qqaq=dirpe0xb9q8qiLsFr0=vr0=vr0dc8meaabaqaciGacaGaaeqabaqabeGadaaakeaacuWGWbaCgaqcamaaDaaaleaacqWGIbGycqWGQbGAaeaacqGGOaakcqaIWaamcqGGPaqkaaGccqGH9aqpcqWGrbqudaahaaWcbeqaaiabgkHiTiabigdaXaaakmaaqahabaGaemiwaG1aaSbaaSqaaiabdMgaPjabdQgaQbqabaaabaGaemyAaKMaeyypa0JaeGymaedabaGaemyuaefaniabggHiLdGccaWLjaGaaCzcamaabmaabaGaeGioaGdacaGLOaGaayzkaaaaaa@4692@

p^aj(0)=p⋅Xsj+p^bj(0)⋅(1−Xsj).     (9)
 MathType@MTEF@5@5@+=feaafiart1ev1aaatCvAUfKttLearuWrP9MDH5MBPbIqV92AaeXatLxBI9gBaebbnrfifHhDYfgasaacH8akY=wiFfYdH8Gipec8Eeeu0xXdbba9frFj0=OqFfea0dXdd9vqai=hGuQ8kuc9pgc9s8qqaq=dirpe0xb9q8qiLsFr0=vr0=vr0dc8meaabaqaciGacaGaaeqabaqabeGadaaakeaacuWGWbaCgaqcamaaDaaaleaacqWGHbqycqWGQbGAaeaacqGGOaakcqaIWaamcqGGPaqkaaGccqGH9aqpcqWGWbaCcqGHflY1cqWGybawdaWgaaWcbaGaem4CamNaemOAaOgabeaakiabgUcaRiqbdchaWzaajaWaa0baaSqaaiabdkgaIjabdQgaQbqaaiabcIcaOiabicdaWiabcMcaPaaakiabgwSixlabcIcaOiabigdaXiabgkHiTiabdIfaynaaBaaaleaacqWGZbWCcqWGQbGAaeqaaOGaeiykaKIaeiOla4IaaCzcaiaaxMaadaqadaqaaiabiMda5aGaayjkaiaawMcaaaaa@533B@

While the sum over query genes to estimate the initial background probability p^bj(0)
 MathType@MTEF@5@5@+=feaafiart1ev1aqatCvAUfKttLearuWrP9MDH5MBPbIqV92AaeXatLxBI9gBaebbnrfifHhDYfgasaacH8akY=wiFfYdH8Gipec8Eeeu0xXdbba9frFj0=OqFfea0dXdd9vqai=hGuQ8kuc9pgc9s8qqaq=dirpe0xb9q8qiLsFr0=vr0=vr0dc8meaabaqaciGacaGaaeqabaqabeGadaaakeaacuWGWbaCgaqcamaaDaaaleaacqWGIbGycqWGQbGAaeaacqGGOaakcqaIWaamcqGGPaqkaaaaaa@339F@ should formally exclude the seed gene, we include it to prevent initialization to zero probability in the case that only the seed gene has an interaction with library gene *j*. The choice of 0 <*p *< 1 in Eq. (9) depends on our confidence about the seed gene's interactions. We have used *p*= 0.95 for the analysis that follows, corresponding to a false positive rate of 5% in the SGA experiment. The motifs extracted are not sensitive to the choice of *p *in the vicinity of the experimental false positive rate (e.g. *p *≥ 0.9). A more detailed discussion on motifs' dependencies on *p *will be given in the DISCUSSION section and it will be made clear that by adjusting *p*, it is possible to systematically retrieve motif members based on the degree of similarity between their genetic interaction patterns and that of the seed. The number of motif members for any seed gene is most likely to be a small portion of the size of the query gene set Q = 126. Thus the number of genes in the motif set is initialized by a^M(0)
 MathType@MTEF@5@5@+=feaafiart1ev1aaatCvAUfKttLearuWrP9MDH5MBPbIqV92AaeXatLxBI9gBaebbnrfifHhDYfgasaacH8akY=wiFfYdH8Gipec8Eeeu0xXdbba9frFj0=OqFfea0dXdd9vqai=hGuQ8kuc9pgc9s8qqaq=dirpe0xb9q8qiLsFr0=vr0=vr0dc8meaabaqaciGacaGaaeqabaqabeGadaaakeaacuWGHbqygaqcamaaDaaaleaacqWGnbqtaeaacqGGOaakcqaIWaamcqGGPaqkaaaaaa@31F9@ ∊ [5,15]. Our analysis shows that the algorithm is not sensitive to the choice of a^M(0)
 MathType@MTEF@5@5@+=feaafiart1ev1aaatCvAUfKttLearuWrP9MDH5MBPbIqV92AaeXatLxBI9gBaebbnrfifHhDYfgasaacH8akY=wiFfYdH8Gipec8Eeeu0xXdbba9frFj0=OqFfea0dXdd9vqai=hGuQ8kuc9pgc9s8qqaq=dirpe0xb9q8qiLsFr0=vr0=vr0dc8meaabaqaciGacaGaaeqabaqabeGadaaakeaacuWGHbqygaqcamaaDaaaleaacqWGnbqtaeaacqGGOaakcqaIWaamcqGGPaqkaaaaaa@31F9@ in this range.

### Results on SGA dataset

**Figure 2:**
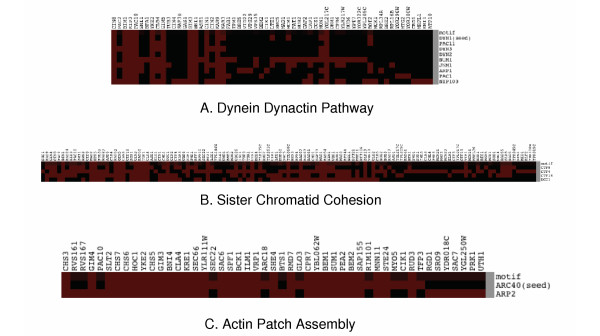
**Representative genetic interaction patterns of the seed, the motif and motif members**. Seed genes for the motifs are (a) DYN1 (b) CTF8 (c) ARC40. The columns correspond to library genes (interaction partners of query genes). Library genes that have no interaction with the seed and the motif members are not shown. Synthetic lethal interactions are represented by red squares. The non-binary values of the motifs are shown by intermediate colors changing from black to red.

Outputs of our model are p^a
 MathType@MTEF@5@5@+=feaafiart1ev1aaatCvAUfKttLearuWrP9MDH5MBPbIqV92AaeXatLxBI9gBaebbnrfifHhDYfgasaacH8akY=wiFfYdH8Gipec8Eeeu0xXdbba9frFj0=OqFfea0dXdd9vqai=hGuQ8kuc9pgc9s8qqaq=dirpe0xb9q8qiLsFr0=vr0=vr0dc8meaabaqaciGacaGaaeqabaqabeGadaaakeaacuWGWbaCgaqcamaaBaaaleaacqWGHbqyaeqaaaaa@2F9E@, p^b
 MathType@MTEF@5@5@+=feaafiart1ev1aaatCvAUfKttLearuWrP9MDH5MBPbIqV92AaeXatLxBI9gBaebbnrfifHhDYfgasaacH8akY=wiFfYdH8Gipec8Eeeu0xXdbba9frFj0=OqFfea0dXdd9vqai=hGuQ8kuc9pgc9s8qqaq=dirpe0xb9q8qiLsFr0=vr0=vr0dc8meaabaqaciGacaGaaeqabaqabeGadaaakeaacuWGWbaCgaqcamaaBaaaleaacqWGIbGyaeqaaaaa@2FA0@ and z^i
 MathType@MTEF@5@5@+=feaafiart1ev1aaatCvAUfKttLearuWrP9MDH5MBPbIqV92AaeXatLxBI9gBaebbnrfifHhDYfgasaacH8akY=wiFfYdH8Gipec8Eeeu0xXdbba9frFj0=OqFfea0dXdd9vqai=hGuQ8kuc9pgc9s8qqaq=dirpe0xb9q8qiLsFr0=vr0=vr0dc8meaabaqaciGacaGaaeqabaqabeGadaaakeaacuWG6bGEgaqcamaaDaaaleaacqWGPbqAaeaaaaaaaa@2FC3@. The motif and background interaction probabilities p^a
 MathType@MTEF@5@5@+=feaafiart1ev1aaatCvAUfKttLearuWrP9MDH5MBPbIqV92AaeXatLxBI9gBaebbnrfifHhDYfgasaacH8akY=wiFfYdH8Gipec8Eeeu0xXdbba9frFj0=OqFfea0dXdd9vqai=hGuQ8kuc9pgc9s8qqaq=dirpe0xb9q8qiLsFr0=vr0=vr0dc8meaabaqaciGacaGaaeqabaqabeGadaaakeaacuWGWbaCgaqcamaaBaaaleaacqWGHbqyaeqaaaaa@2F9E@ and p^b
 MathType@MTEF@5@5@+=feaafiart1ev1aaatCvAUfKttLearuWrP9MDH5MBPbIqV92AaeXatLxBI9gBaebbnrfifHhDYfgasaacH8akY=wiFfYdH8Gipec8Eeeu0xXdbba9frFj0=OqFfea0dXdd9vqai=hGuQ8kuc9pgc9s8qqaq=dirpe0xb9q8qiLsFr0=vr0=vr0dc8meaabaqaciGacaGaaeqabaqabeGadaaakeaacuWGWbaCgaqcamaaBaaaleaacqWGIbGyaeqaaaaa@2FA0@ are two position weight matrices with continuous elements in the range of [0,1]. Unlike in the case of DNA binding site detection, the converged probabilities z^i
 MathType@MTEF@5@5@+=feaafiart1ev1aaatCvAUfKttLearuWrP9MDH5MBPbIqV92AaeXatLxBI9gBaebbnrfifHhDYfgasaacH8akY=wiFfYdH8Gipec8Eeeu0xXdbba9frFj0=OqFfea0dXdd9vqai=hGuQ8kuc9pgc9s8qqaq=dirpe0xb9q8qiLsFr0=vr0=vr0dc8meaabaqaciGacaGaaeqabaqabeGadaaakeaacuWG6bGEgaqcamaaDaaaleaacqWGPbqAaeaaaaaaaa@2FC3@ computed for the SGA dataset are either very close to 0 or very close to 1; intermediate values have not been observed. Thus, given a seed gene, the remaining genes are naturally categorized as motif genes (with *z_*i *_*≈ 1) or non-motif genes (with *z_*i *_*≈ 0). We call motif genes motif members of the seed. Three representative motifs are shown along with the genetic interaction patterns of the seed genes *DYN1*, *CTF8 *and *ARC40 *and their motif members (Fig. 2). Table 1 shows the groups of motif members obtained for seed genes *ARL1*, *SKT5*, *CTF8 *and *RIC1 *when various values of *p *are used. Motif members obtained with *p *= 0.95 for 13 seed genes are listed in Table 2. The full table is available as supporting material (additional file gimf-motifs.txt). The seven groups of genes thus identified agree with groups observed with hierarchical clustering [12], which supports GIMF's capacity in extracting biologically relevant gene pathways.

**Table 1:** Motif members of four seed genes.

Seed	Motif members			
	
	*p *= 0.6	*p *= 0.7	*p = 0.8*	*p *= 0.95
ARL1	ARL3, SWF1, RIC1, YPT6	RIC1, YPT6, ARL3		
SKT5	CHS6, CHS3, CHS7, CHS5	CHS3, CHS7, CHS5		CHS3, CHS5
CTF8	CTF4, CTF18, DCC1, BIM1, CIN8, KAR3	CTF4, CTF18 DCC1, BIM1	CTF4, CTF18, DCC1	CTF4, CTF18, DCC1
RIC1	YPT6			

Motif members of ARL1, SKT5, CTF8 and RIC1 are obtained by choosing different values of initialization parameter *p*. Most motifs show little dependency on p for p ≥ 0.9.

**Table 2:** Seven representative motifs identified by GIMF.

	Pathway or complex	Seed gene	Motif gene list
1	Actin patch assembly	ARC40	ARP2
2	Chitin synthase III pathway	CHS7	CHS3, SKT5, CHS5
		CHS6	CHS3, SKT5
3	Prefoldin complex	PAC10	GIM3, GIM4, GIM5, YKE2
4	Membrane traffic	ARL1	ARL3, RIC1, YPT6
		GYP1	RIC1
5	Dynein Dynactin pathway	DYN1	ARP1, DYN1, PAC11, YMR299C, DYN2, JNM1, PAC1, NIP100, NUM1
		PAC1	ARP1, DYN1, PAC11, YMR299C, DYN2, JNM1, NIP100, NUM1
		JNM1	ARP1, DYN1, PAC11, YMR299C, DYN2, NIP100, NUM1
		NUM1	JNM1
6	DNA replication checkpoint	MRC1	TOF1
7	Sister chromatid cohesion	DCC1	CTF4, CTF18
		CTF8	CTF18, DCC1, CTF4

These gene modules correlate well with pathways inferred by hierarchical clustering (Tong et al. 2004).

**Figure 3:**
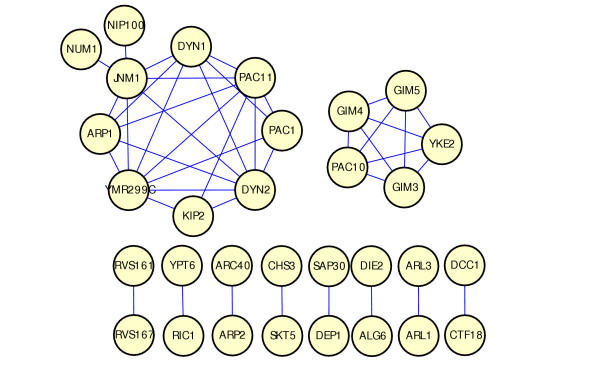
**GIMF Type 1 network**. The network is created by applying Rule 1 to the motif member lists of all query genes. The network contains 31 nodes and 42 edges, where the nodes are query genes and an edge between node *i *and node *j *indicates that *i *and *j *are each other's motif members.

One important property of GIMF is that it is non-commutative: if gene A identifies gene B as a motif member, it is not necessarily true that gene B identifies gene A as its motif member. Interestingly, we have observed that a seed gene tends to first pull up motif members that share a globally similar interaction pattern. If such genes are lacking, then it finds genes with locally similar interaction pattern. This enables us to probe the case when two genes' interaction partners are only similar on a local scale. This is not possible with pair-wise comparison metrics, which are commutative. For a more systematic analysis, we use GIMF to build gene networks. First, query genes with very few interactions (5 or fewer) are removed from the list of seeds. Then each of the remaining query genes is used as a seed and its motif members are generated by GIMF. For every query gene pair(*i, j*), if *i *and *j *are each other's motif member, then connect *i *and *j *with a Type 1 edge. We call the network thus constructed a Type 1 GIMF network (Fig. 3). This network contains 31 nodes and 42 edges, which form two clusters and eight individual pairs. The smaller cluster is a fully connected sub-graph corresponding to the *PAC10 *complex. The larger cluster with 10 genes (*ARP1, NUM1, DYN1, PAC11, PAC1, DYN2, JNM1, YMR299C, NIP100, KIP2*), representing the Dynein-Dynactin spindle orientation pathway. *KIP2 *was not detected by hierarchical clustering [12].

**Figure 4:**
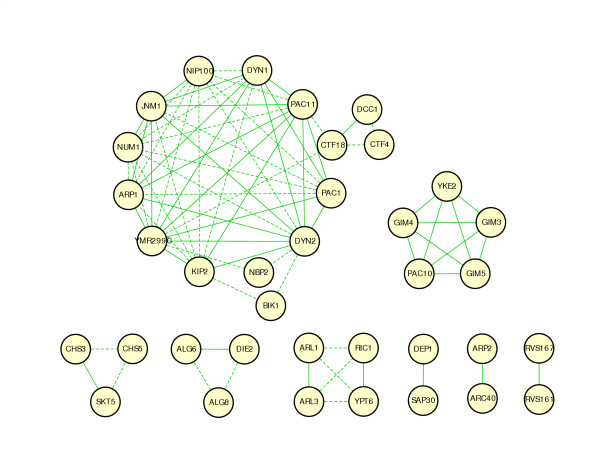
**GIMF Type 2 network**. This network is created by applying Rule 2 to all the edges in the GIMF Type 1 network. See text for details. The solid edges are inherited from the Type 1 network while the dashed edges are added by applying Rule 2.

Apparently, the bi-directional rule only retains genes with globally similar interaction pattern. This can be quite stringent since genes have multiple functions and two genes operating in one pathway may have distinct roles in other pathways they participate and thus only share a fraction of synthetic lethal interaction partners. Thus we extend Type 1 network by the following simple rule: for each gene pair (*i, j*) in the Type 1 network, add common motif members *k *of genes *i *and *j *that are not already in the network (hence neither *i *nor *j *is motif member of *k*). Connect *k *to *i *and *j *with a Type 2 edge. We call the extended network a Type 2 network (Fig. 4). This analysis reveals more information in the *Dynein-Dynactin *pathway. The majority of Type 2 edges occur between the group members of this cluster, which elevates the confidence that the genes within this cluster are closely related. Evidence that genes in this cluster are biologically related include the presence of a dynactin protein complex (*ARP1, JNM1, NIP100*), reported protein-protein interactions between *NIP100-PAC11, PAC11-DYN2, PAC11-NUM1 *[22,23] and the suggestion that *YMR299C *functions as dynein light intermediate chain [12]. In the Type 2 network, several new members are incorporated into the cluster, including *NBP2, BIK1 *and *CTF18*. The molecular function of *NBP2 *and *CTF18 *are unknown while *BIK1 *is involved in microtubule binding. *NBP2 *shows hyperosmotic and heat response and is a negative regulator of protein kinase activity. *CTF18 *is a subunit of a complex with *CTF8P *that shares some subunits with Replication Factor C and is required for sister chromatid cohesion. It has been known that the mutants of six genes (*NUM1, DYN1, DYN2, ARP1, JNM1, NIP100*) in this cluster show nuclear migration defect in cell division process. A recent experiment has confirmed that deletion mutants of *KIP2, BIK1 *and *CTF18 *also exhibit moderate to severe nuclear migration defects [24]. These three genes have not been detected by two way clustering [12].

Under our hypothesis, genes with a similar synthetic interaction pattern (especially when the similarity is global) are likely to reside in the same pathway or map to proteins in the same complex. Thus the motif members are expected to have functional similarities at various levels. We evaluate the biological relevance of the Type 1 and Type 2 networks by computing three parameters for each edge (gene pair): the correlations with the Gene Ontology (GO) annotations (described in Appendix); the fraction of gene products that are within the same protein complex as determined by high-throughput mass spectrometry; and the fraction that are synthetic lethal. These parameters have also been computed for all directly synthetic lethal gene pairs. The Type 1 gene pairs' correlations for biological process, molecular function and cellular component GO annotations are (0.47, 0.20, 0.43), while those of the Type 2 network are (0.47, 0.15, 0.40), comparing to (0.25, 0.05, 0.31) for directly synthetic lethal gene pairs (Table 3). Clearly, much tighter functional associations are obtained between gene pairs with either globally or locally similar synthetic lethal interactions than gene pairs that are directly synthetic lethal interactions, confirming the observation of between-pathway enrichment by Wong et al. and Kelly et al. [13,14]. Significantly more Type 1 gene pairs map to proteins within the same complex than either Type 2 gene pairs or directly synthetic lethal gene pairs. Same-complex membership may explain the higher molecular function correlation for Type 1 gene pairs.

**Table 3:** GO annotation correlations for GIMF Type 1, Type 2 gene pairs, and gene pairs that are directly synthetic lethal (SL).

Gene pairs	GO correlation			FSL	FPC	Number of pairs	Number of genes
					
	P	F	C				
Type 1	0.47	0.20	0.43	0	0.26	42	31
Type 2	0.47	0.15	0.40	0.07	0.14	78	36
SL	0.25	0.05	0.31	--	0.01	3474	1004

P: biological process; F: molecular function C: cellular component. FSL: fraction of pairs that are directly synthetic lethality; FPC: fraction of pairs that are within the same protein complex. The GIMF Type 1 network contains 31 genes and 42 edges while the Type 2 network contains 36 genes and 78 edges.

## Discussion

In this section, we explore a few important issues in terms of the robustness and tuning of GIMF. Without loss of generality, the discussion is primarily based on learning pathway association on the SGA dataset.

It has been widely known that EM algorithm very often converges to local maxima in the evaluation of posterior likelihood function or log-likelihood [18,19]. In application to motif (e.g. transcription binding sites) discovery in DNA sequences, early versions EM assumed the existence of a single motif and aimed to find the motif that globally optimized the likelihood function. However, when multiple consensus sequences are present in the dataset, numerous local maxima in the likelihood function can well correspond to biologically meaningful motifs. One approach to finding multiple motifs is to initialize the EM from different starting points, typically selected from patterns occurring in the data, which may then relax to local maxima. This approach may be enhanced, as in the MEME algorithm, by erasing motifs previously found so that multiple motifs are found in decreasing order of likelihoods. Using these two strategies, MEME successfully detects multiple promoter consensuses from the combined CRP/LexA datasets[18].

In GIMF, we achieve a similar effect by initializing the model using seed gene's interactions, thus narrowing down the search space to the module that includes the seed. Without any prior knowledge of goodness of seeds and their consensus interactions, two problems are noteworthy: i) Motifs generated by different seeds may be redundant; ii) Certain motifs may deviate from their seeds during the iterative process. These two issues are addressed below:

### i) Motifs generated by different seeds may be redundant

**Figure 5:**
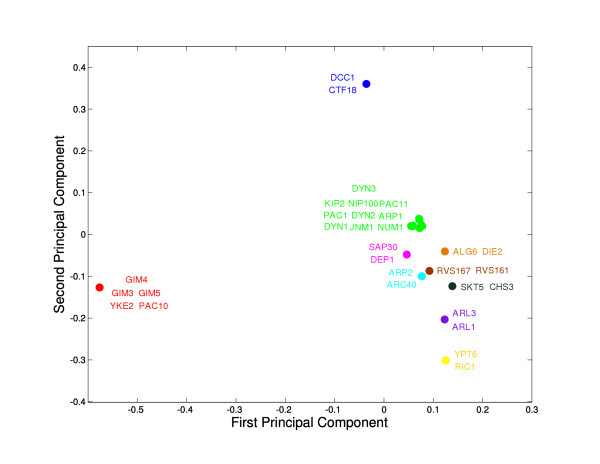
**Two-dimensional embedding of Type 1 network motifs**. Each motif is represented by two leading principal components generated by multidimensional scaling based on a Euclidean distance matrix. Colors indicate connected components from the Type 1 network.

To better understand the dissimilarity between distinct motifs, we have calculated the Euclidean distance between each pair of motifs p^alS1
 MathType@MTEF@5@5@+=feaafiart1ev1aqatCvAUfKttLearuWrP9MDH5MBPbIqV92AaeXatLxBI9gBaebbnrfifHhDYfgasaacH8akY=wiFfYdH8Gipec8Eeeu0xXdbba9frFj0=OqFfea0dXdd9vqai=hGuQ8kuc9pgc9s8qqaq=dirpe0xb9q8qiLsFr0=vr0=vr0dc8meaabaqaciGacaGaaeqabaqabeGadaaakeaacuWGWbaCgaqcamaaDaaaleaacqWGHbqycqWGSbaBaeaacqWGtbWucqaIXaqmaaaaaa@3320@ and p^alS2
 MathType@MTEF@5@5@+=feaafiart1ev1aqatCvAUfKttLearuWrP9MDH5MBPbIqV92AaeXatLxBI9gBaebbnrfifHhDYfgasaacH8akY=wiFfYdH8Gipec8Eeeu0xXdbba9frFj0=OqFfea0dXdd9vqai=hGuQ8kuc9pgc9s8qqaq=dirpe0xb9q8qiLsFr0=vr0=vr0dc8meaabaqaciGacaGaaeqabaqabeGadaaakeaacuWGWbaCgaqcamaaDaaaleaacqWGHbqycqWGSbaBaeaacqWGtbWucqaIYaGmaaaaaa@3322@ generated by seed S1 and S2, respectively, resulting in a 126 by 126 distance matrix *D*. To visualize this matrix, we embed it in two dimensions using classic multidimensional scaling, which is essentially equivalent to projecting the two leading principal components. Motifs corresponding to connected components in the Type 1 network are close together in this embedding (Fig. 5). In most cases, the seed genes in a Type 1 connected component have either overlapping motifs (several motifs collapse onto one point) or motifs that are very similar to each other. In comparison with the greater number of maxima identified for all query genes (Fig. S2), this analysis suggests that the local maxima corresponding to queries in the Type 1 network are reproducibly identified. These local maxima could be considered global maxima conditioned on the seed gene remaining in the motif.

### ii) Certain motifs may deviate from their seeds during the iterative process

In some cases, the EM algorithm may eject a seed gene from a motif. This occurs for eight seed genes when *p *= 0.95 using the threshold *Z*_*i *_> 0.9 (Table S2). Those seeds either have few interactions and/or have interactions that overlap largely with the interaction partners of some hub genes, such as the *PAC10 *complex genes. Indeed, most of their motif members are hub genes, whose interaction profiles override that of the seed genes during the iteration. Thus to ensure each seed stay in the motif, we can slightly modify the algorithm by fixing *Z*_*seed *_= 1 during all iterations. In other words, the motif search is conditioned on the seed being part of the motif. Indeed, for the eight seeds mentioned above, this modification keeps the seed gene itself in the motif till convergence while all other motif members stay unchanged. Clearly, this procedure has no effect on the 106 seeds that are already in motif without such conditioning.

Symmetry imposed by Type 1 edges serves as a conservative filtering procedure that eliminates redundancy and impact of hub genes dominating interaction profiles, which reveals gene networks with tight functional correlations, which supports our finding that the local optimums in GIMF correspond to biologically relevant modules.

We have investigated how the choice of *p*, the initialization parameter that represents our confidence on seed gene's interactions, affect the motifs. Indeed, the sensitivities of different motifs to *p *is non-uniform. We quantify the goodness of a seed and its motif by observing stability of its motif members across different choices of *p*. Genes with less than five interaction partners (12 out of 126) are not used as seeds. For every remaining query gene, we extract its motif members with *p *ranging from 0.6 to 0.95. The sets of motif members extracted at *p = 0.95 *is used as the reference to compute a Jaccard coefficient [14]. Denote the set of motif members for seed gene *i *obtained with initialization parameter *p *by Mpi
 MathType@MTEF@5@5@+=feaafiart1ev1aqatCvAUfKttLearuWrP9MDH5MBPbIqV92AaeXatLxBI9gBaebbnrfifHhDYfgasaacH8akY=wiFfYdH8Gipec8Eeeu0xXdbba9frFj0=OqFfea0dXdd9vqai=hGuQ8kuc9pgc9s8qqaq=dirpe0xb9q8qiLsFr0=vr0=vr0dc8meaabaqaciGacaGaaeqabaqabeGadaaakeaacqWGnbqtdaqhaaWcbaGaemiCaahabaGaemyAaKgaaaaa@30C3@. The corresponding Jaccard coefficient Jpi
 MathType@MTEF@5@5@+=feaafiart1ev1aaatCvAUfKttLearuWrP9MDH5MBPbIqV92AaeXatLxBI9gBaebbnrfifHhDYfgasaacH8akY=wiFfYdH8Gipec8Eeeu0xXdbba9frFj0=OqFfea0dXdd9vqai=hGuQ8kuc9pgc9s8qqaq=dirpe0xb9q8qiLsFr0=vr0=vr0dc8meaabaqaciGacaGaaeqabaqabeGadaaakeaacqWGkbGsdaqhaaWcbaGaemiCaahabaGaemyAaKgaaaaa@30BC@ is given by Jpi=Mpi∩Mp=0.95iMpi∪Mp=0.95i
 MathType@MTEF@5@5@+=feaafiart1ev1aaatCvAUfKttLearuWrP9MDH5MBPbIqV92AaeXatLxBI9gBaebbnrfifHhDYfgasaacH8akY=wiFfYdH8Gipec8Eeeu0xXdbba9frFj0=OqFfea0dXdd9vqai=hGuQ8kuc9pgc9s8qqaq=dirpe0xb9q8qiLsFr0=vr0=vr0dc8meaabaqaciGacaGaaeqabaqabeGadaaakeaacqWGkbGsdaqhaaWcbaGaemiCaahabaGaemyAaKgaaOGaeyypa0ZaaSaaaeaacqWGnbqtdaqhaaWcbaGaemiCaahabaGaemyAaKgaaOGaeyykICSaemyta00aa0baaSqaaiabdchaWjabg2da9iabicdaWiabc6caUiabiMda5iabiwda1aqaaiabdMgaPbaaaOqaaiabd2eannaaDaaaleaacqWGWbaCaeaacqWGPbqAaaGccqGHQicYcqWGnbqtdaqhaaWcbaGaemiCaaNaeyypa0JaeGimaaJaeiOla4IaeGyoaKJaeGynaudabaGaemyAaKgaaaaaaaa@4F28@. The seeds can be divided into four categories based on their Jaccard coefficient averaged over the different values of *p*: i)J¯i
 MathType@MTEF@5@5@+=feaafiart1ev1aaatCvAUfKttLearuWrP9MDH5MBPbIqV92AaeXatLxBI9gBaebbnrfifHhDYfgasaacH8akY=wiFfYdH8Gipec8Eeeu0xXdbba9frFj0=OqFfea0dXdd9vqai=hGuQ8kuc9pgc9s8qqaq=dirpe0xb9q8qiLsFr0=vr0=vr0dc8meaabaqaciGacaGaaeqabaqabeGadaaakeaacuWGkbGsgaqeamaaCaaaleqabaGaemyAaKgaaaaa@2F6B@ = 1. Those seeds have invariant motif members and are hence categorized as very strong seeds. Special cases are seeds that exact only themselves. Those seeds have unique interaction patterns but most likely do not have any pathway members included in the query set. ii)0.9 <J¯i
 MathType@MTEF@5@5@+=feaafiart1ev1aaatCvAUfKttLearuWrP9MDH5MBPbIqV92AaeXatLxBI9gBaebbnrfifHhDYfgasaacH8akY=wiFfYdH8Gipec8Eeeu0xXdbba9frFj0=OqFfea0dXdd9vqai=hGuQ8kuc9pgc9s8qqaq=dirpe0xb9q8qiLsFr0=vr0=vr0dc8meaabaqaciGacaGaaeqabaqabeGadaaakeaacuWGkbGsgaqeamaaCaaaleqabaGaemyAaKgaaaaa@2F6B@ < 1. These are strong seeds with almost invariant motif members. iii)0.6 <J¯i
 MathType@MTEF@5@5@+=feaafiart1ev1aaatCvAUfKttLearuWrP9MDH5MBPbIqV92AaeXatLxBI9gBaebbnrfifHhDYfgasaacH8akY=wiFfYdH8Gipec8Eeeu0xXdbba9frFj0=OqFfea0dXdd9vqai=hGuQ8kuc9pgc9s8qqaq=dirpe0xb9q8qiLsFr0=vr0=vr0dc8meaabaqaciGacaGaaeqabaqabeGadaaakeaacuWGkbGsgaqeamaaCaaaleqabaGaemyAaKgaaaaa@2F6B@ < 0.9. This case corresponds to moderately strong seeds whose motif members change moderately and hierarchically. Decreasing *p *decreases the confidence in the seed gene's interactions, and genes with more distant interaction profiles can be incorporated into the motif set. The motif members converge at confidence level close to the true experimental false positive rate. iv)J¯i
 MathType@MTEF@5@5@+=feaafiart1ev1aaatCvAUfKttLearuWrP9MDH5MBPbIqV92AaeXatLxBI9gBaebbnrfifHhDYfgasaacH8akY=wiFfYdH8Gipec8Eeeu0xXdbba9frFj0=OqFfea0dXdd9vqai=hGuQ8kuc9pgc9s8qqaq=dirpe0xb9q8qiLsFr0=vr0=vr0dc8meaabaqaciGacaGaaeqabaqabeGadaaakeaacuWGkbGsgaqeamaaCaaaleqabaGaemyAaKgaaaaa@2F6B@ < 0.6. Those seeds have highly variable motif members and hence are weak seeds. The numbers of seeds in the four categories are 14, 6, 45 and 49, respectively (Table S1). When analyzing the SGA dataset, *p *= 0.95 is reasonable because interactions in the SGA dataset have been experimentally validated and has a low false-positive rate. This analysis has three important indications: i) Not all the query genes are good seeds, partly due to the incompleteness of the synthetic lethal genetic interaction map in the query axis; ii) To achieve optimal detection of motifs for different seeds, we might need to employ different initialization parameter *p*. Given a minimum Jaccard coefficient, the algorithm can be optimally initialized for each seed. iii) The Type 1 network obtained at *p = 0.95 *is most likely conservative. Thus to build gene networks with better confidence, we may eliminate bad seeds, relax confidence constraints on strong and moderately strong seeds while imposing an initialization parameter close to 1 on weak seeds.

To better evaluate the statistical significance of motifs detected by GIMF, we have computed the false positive rates on randomized datasets with the same degree distribution as the original synthetic lethal dataset. Randomization is done by a rewiring procedure as detailed in [21]. The fraction of overlapping links between the randomized network and the original network is around 15%. Since a random network should not contain any biologically relevant motif, any motif detected is a false positive. Thus for the GIMF algorithm, we use every query as a seed gene and any motif member returned other than the seed itself is considered a false positive. The numbers of false positives on 100 randomized networks are shown in Fig. S1. Without imposing the bi-directionality constraint, the average total number of false positives for 126 seed genes is 15. The average number of seeds that generates any false positives is 9.7 out of 126. On the real dataset, the number of seeds leading to motif detection is *T *= 82. Thus this corresponds to a p-value of 10^-15 ^calculated as tail probability at *T *= 82 from a Poisson distribution. A detailed look at the false positive pairs of GIMF shows that most seeds that lead to false positives have very few interactions with the library genes. The top 10 seeds producing the most false positives have 6.3 interactions on average and their false positive motif genes are mostly promiscuous hub genes. However, no false positives are observed when the promiscuous genes are used as seeds. When bi-directionality is imposed on motif detection, false positive drops to 0 for all the 100 trials. Thus for an asymmetric metric like GIMF, we can impose symmetry constraint to mask the effects of promiscuous genes. Additional information can be obtained by elevating stringency once the reliable gene pairs are identified.

The treatment of hub genes is a problematic issue in the analysis of power-law networks. Hubs arise from many different sources including intrinsic error in the experimental technique (such as sticky proteins in yeast two hybrid system) and experimental bias (such as the choice of query genes for SGA). Because of this heterogeneity, the treatment of promiscuous genes should be context-based. In the case of experimental error-induced hubs, a straightforward approach is to ignore all hub-associated links. This filtering method has been used to reduce the number of candidate pathways dramatically in the analysis of signal transduction networks [25]. However, the role of hub genes in the SGA data set is subtle. Genes in the *PAC10 *complex are hubs that have enriched synthetic lethal interaction with genes in many other complexes, such as *CTF18 *and *PAC11*. Many of the *PAC10*-associated links are biologically relevant since *PAC10 *is indeed functionally coupled to a broad spectrum of biological pathways which themselves are functionally associated. Thus removing hub links entirely unsurprisingly leads to the loss of useful information and failure to detect some relevant pathways. GIMF treats this problem by permitting an increase in the parameter *p*_*bj *_for hub library genes that are not part of the motif. Thus, we are able to extract biologically meaningful pathways by keeping the hub library genes whose impact is, however, automatically down-weighted.

This idea is tested on the Dynein-Dynactin gene pairs. Using GIMF we identified 24 Dynein-Dynactin pairs with the original SGA dataset. Then we tested GIMF on five filtered datasets generated by removing interactions with the top 5, 10, 15, 20 and 25 hub library genes, respectively. The corresponding fractions of interaction eliminated are 4.4%, 7.8%, 10.9%, 13.6% and 16%. With model parameters unchanged, GIMF recovers (18, 15, 10, 7, 1) Dynein-Dynactin pairs on the five datasets, respectively. The reduced coverage is expected from the removal of some biologically relevant hub links. However, a substantial number of those pairs are retained when interactions with the top 5 and 10 hub library genes are absent. These results suggest that a statistical method that explicitly models the skewed degree distribution is a better strategy for pattern discovery in the presence of hubs than using simple filtering techniques in conjunction with methods that do not take into account the hub effect.

The assumption in GIMF that the probability of an edge between a query and a library gene pair is proportional to the degree of the library gene works sufficiently well for the synthetic lethal interaction dataset. However, when extending the present model to other types of networks especially those with non-directional links, it would be beneficial to characterize the link probability in a subgraph based on local connectivities [26]. In this model, the link probability between a pair of genes depends on the degree of both genes. This allows us to consider each interaction in the context of its subgraph, thus has a good promise to extract motifs in power-law networks by their local deviations from randomness [27]. It would be interesting to integrate the local models into our algorithm in motif extraction of other interaction networks such as protein interaction networks.

Recently, Kelley et al. have integrated physical protein-protein interactions to dissect synthetic lethal gene pairs into between-pathway and within-pathway paradigms [14]. While the focus of our study is different from their work, GIMF has an interesting correspondence with their algorithm. The algorithm proposed by Kelley et al. to construct between-pathway or within-pathway model is essentially a local search procedure described by Sharan et al [28]. Starting from a seed node, nodes whose contributions to the current seed are maximal are added one at a time. The operation is repeated in a breadth-first search fashion so long as it increases the overall score of the subgraph. This is equivalent to maintaining a set of motif and non-motif nodes each with probability 1 and only the interaction between directly linked nodes are considered during the iteration. In contrast, GIMF maintains a probability of being in the motif set for each node, thus allowing all nodes to have contribution in each iteration during motif building. The assignment of a node to the motif versus non-motif category is only determined when the probabilities converge. A similar breadth-first search procedure can also be applied to GIMF in automatically extracting gene pathways. The between-pathway and within-pathway discovery by Kelley et al. [14] aligns with the conclusion by Tong et. al [12] that synthetic lethal interactions are more abundant between genes that have the same mutant phenotype and the genes encoding proteins within the same protein complex.

**Figure 6:**
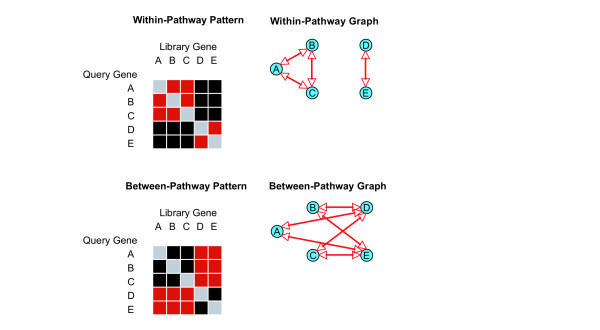
**Within-pathway and between-pathway patterns**. Partial interaction profiles are shown for five query genes where "interaction", "no-interaction", "prohibited self-interaction" are represented by red, black and grey in the interaction matrix respectively. Genes A, B, C and D, E belong to two different pathways. Interactions involving members of the same pathway are enriched in the within-pathway model and depleted in the between-pathway model.

This idea is illustrated in Fig. 6, where "interaction", "no-interaction", "prohibited self-interaction" are represented by red, black and grey respectively. The matrix shows partial interaction profiles for five query genes. Query genes A, B, C and D, E belong to two different motifs. The within-pathway pattern shows the situation where synthetic lethal interactions are more abundant between motif members than between genes belonging to different motifs. The between-pathway pattern shows two motifs that represent two complementary pathways, with synthetic lethal interactions enriched between the pathways and depleted within a pathway. To permit a quantitative discussion, we define a within-motif score (WMS) to characterize whether synthetic lethal interactions for motif genes with each other are enriched (corresponding to the within-pathway pattern) or depleted (corresponding to the between-pathway pattern). Let *WMS*_*i *_represent the score for motif *i *given by

WMSi=∑j=1Q=126Zjilog⁡(fji/bji)/∑j=1Q=126Zji     (10)
 MathType@MTEF@5@5@+=feaafiart1ev1aqatCvAUfKttLearuWrP9MDH5MBPbIqV92AaeXatLxBI9gBaebbnrfifHhDYfgasaacH8akY=wiFfYdH8Gipec8Eeeu0xXdbba9frFj0=OqFfea0dXdd9vqai=hGuQ8kuc9pgc9s8qqaq=dirpe0xb9q8qiLsFr0=vr0=vr0dc8meaabaqaciGacaGaaeqabaqabeGadaaakeaacqWGxbWvcqWGnbqtcqWGtbWudaWgaaWcbaGaemyAaKgabeaakiabg2da9maalyaabaWaaabCaeaacqWGAbGwdaqhaaWcbaGaemOAaOgabaGaemyAaKgaaOGagiiBaWMaei4Ba8Maei4zaCMaeiikaGIaemOzay2aa0baaSqaaiabdQgaQbqaaiabdMgaPbaakiabc+caViabdkgaInaaDaaaleaacqWGQbGAaeaacqWGPbqAaaGccqGGPaqkaSqaaiabdQgaQjabg2da9iabigdaXaqaaiabdgfarjabg2da9iabigdaXiabikdaYiabiAda2aqdcqGHris5aaGcbaWaaabCaeaacqWGAbGwdaqhaaWcbaGaemOAaOgabaGaemyAaKgaaaqaaiabdQgaQjabg2da9iabigdaXaqaaiabdgfarjabg2da9iabigdaXiabikdaYiabiAda2aqdcqGHris5aaaakiaaxMaacaWLjaWaaeWaaeaacqaIXaqmcqaIWaamaiaawIcacaGLPaaaaaa@6461@

where

fji=(Number of interactions with members of motif i) +1(Total number of members of motif i)−Zji+ 1 andbji=(Number of interaction partners)+1(Total number of possible interaction partners)+1.     (11)
 MathType@MTEF@5@5@+=feaafiart1ev1aqatCvAUfKttLearuWrP9MDH5MBPbIqV92AaeXatLxBI9gBaebbnrfifHhDYfgasaacH8akY=wiFfYdH8Gipec8Eeeu0xXdbba9frFj0=OqFfea0dXdd9vqai=hGuQ8kuc9pgc9s8qqaq=dirpe0xb9q8qiLsFr0=vr0=vr0dc8meaabaqaciGacaGaaeqabaqabeGadaaakqaabeqaaiabdAgaMnaaDaaaleaacqWGQbGAaeaacqWGPbqAaaGccqGH9aqpdaWcaaqaaiabbIcaOiabb6eaojabbwha1jabb2gaTjabbkgaIjabbwgaLjabbkhaYjabbccaGiabb+gaVjabbAgaMjabbccaGiabbMgaPjabb6gaUjabbsha0jabbwgaLjabbkhaYjabbggaHjabbogaJjabbsha0jabbMgaPjabb+gaVjabb6gaUjabbohaZjabbccaGiabbEha3jabbMgaPjabbsha0jabbIgaOjabbccaGiabb2gaTjabbwgaLjabb2gaTjabbkgaIjabbwgaLjabbkhaYjabbohaZjabbccaGiabb+gaVjabbAgaMjabbccaGiabb2gaTjabb+gaVjabbsha0jabbMgaPjabbAgaMjabbccaGiabdMgaPjabcMcaPiabbccaGiabbUcaRiabbgdaXaqaaiabbIcaOiabbsfaujabb+gaVjabbsha0jabbggaHjabbYgaSjabbccaGiabb6gaUjabbwha1jabb2gaTjabbkgaIjabbwgaLjabbkhaYjabbccaGiabb+gaVjabbAgaMjabbccaGiabb2gaTjabbwgaLjabb2gaTjabbkgaIjabbwgaLjabbkhaYjabbohaZjabbccaGiabb+gaVjabbAgaMjabbccaGiabb2gaTjabb+gaVjabbsha0jabbMgaPjabbAgaMjabbccaGiabdMgaPjabcMcaPiabgkHiTiabdQfaAnaaDaaaleaacqWGQbGAaeaacqWGPbqAaaGccqGHRaWkcqqGGaaicqaIXaqmaaGaeeiiaaIaeeyyaeMaeeOBa4MaeeizaqgabaGaemOyai2aa0baaSqaaiabdQgaQbqaaiabdMgaPbaakiabg2da9maalaaabaGaeeikaGIaeeOta4KaeeyDauNaeeyBa0MaeeOyaiMaeeyzauMaeeOCaiNaeeiiaaIaee4Ba8MaeeOzayMaeeiiaaIaeeyAaKMaeeOBa4MaeeiDaqNaeeyzauMaeeOCaiNaeeyyaeMaee4yamMaeeiDaqNaeeyAaKMaee4Ba8MaeeOBa4MaeeiiaaIaeeiCaaNaeeyyaeMaeeOCaiNaeeiDaqNaeeOBa4MaeeyzauMaeeOCaiNaee4CamNaeiykaKIaey4kaSIaeGymaedabaGaeeikaGIaeeivaqLaee4Ba8MaeeiDaqNaeeyyaeMaeeiBaWMaeeiiaaIaeeOBa4MaeeyDauNaeeyBa0MaeeOyaiMaeeyzauMaeeOCaiNaeeiiaaIaee4Ba8MaeeOzayMaeeiiaaIaeeiCaaNaee4Ba8Maee4CamNaee4CamNaeeyAaKMaeeOyaiMaeeiBaWMaeeyzauMaeeiiaaIaeeyAaKMaeeOBa4MaeeiDaqNaeeyzauMaeeOCaiNaeeyyaeMaee4yamMaeeiDaqNaeeyAaKMaee4Ba8MaeeOBa4MaeeiiaaIaeeiCaaNaeeyyaeMaeeOCaiNaeeiDaqNaeeOBa4MaeeyzauMaeeOCaiNaee4CamNaeiykaKIaey4kaSIaeGymaedaaiabc6caUiaaxMaacaWLjaWaaeWaaeaacqaIXaqmcqaIXaqmaiaawIcacaGLPaaaaaaa@1D99@

The total number of motif members is the denominator of Eq. 10, and the add-one pseudocounts in fji
 MathType@MTEF@5@5@+=feaafiart1ev1aqatCvAUfKttLearuWrP9MDH5MBPbIqV92AaeXatLxBI9gBaebbnrfifHhDYfgasaacH8akY=wiFfYdH8Gipec8Eeeu0xXdbba9frFj0=OqFfea0dXdd9vqai=hGuQ8kuc9pgc9s8qqaq=dirpe0xb9q8qiLsFr0=vr0=vr0dc8meaabaqaciGacaGaaeqabaqabeGadaaakeaacqWGMbGzdaqhaaWcbaGaemOAaOgabaGaemyAaKgaaaaa@30E9@ and bji
 MathType@MTEF@5@5@+=feaafiart1ev1aqatCvAUfKttLearuWrP9MDH5MBPbIqV92AaeXatLxBI9gBaebbnrfifHhDYfgasaacH8akY=wiFfYdH8Gipec8Eeeu0xXdbba9frFj0=OqFfea0dXdd9vqai=hGuQ8kuc9pgc9s8qqaq=dirpe0xb9q8qiLsFr0=vr0=vr0dc8meaabaqaciGacaGaaeqabaqabeGadaaakeaacqWGIbGydaqhaaWcbaGaemOAaOgabaGaemyAaKgaaaaa@30E1@ bound the output of the log transform. The more negative an WMS, the more a motif reflects between-pathway interactions.

**Figure 7:**
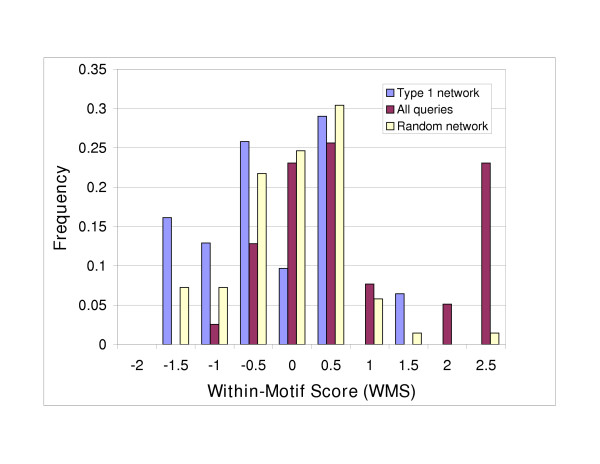
**Within-Pathway Score (WMS) probability distributions**. Distributions of the WMS on motifs from Type 1 network set (blue), all query set (red) and random network set (yellow). The WMS mean ± standard error for the Type 1 network is -0.250 ± 0.055; all query set, -0.011 ± 0.089 ; random network, 0.91 ± 0.21

The WMS was computed for three sets of motifs generated: i) for seeds in the Type 1 network; ii) for all seeds from the query set; iii) for seeds in 100 randomized datasets described earlier (Fig. 7). The distribution of WMS values for motifs in the actual network appears bimodal, with greater probability for motifs with between-pathway character (WMS < 0). The WMS distribution for the Type 1 network has significantly more between-pathway character compared to motifs discovered in random network (one-sided, unequal variance t-test on WMS values, p-value = 1.4 × 10^-5^). Motifs in the entire network also have significantly more between-pathway character, as judged by smaller WMS values, than motifs in the random network (p-value = 6.6 × 10^-5^). Motifs from the Type 1 network show marginal significance for negative WMS values (one-sided z-test, p-value = 0.055), whereas motifs from the random network have significantly positive WMS values (p-value 4.5 × 10^-6^). In summary, these results demonstrate that synthetic lethal interactions leading to motifs have significant between-pathway character, particularly when compared with motifs detected in randomized networks.

Though the purpose of this study is to develop a probabilistic model for characterizing synthetic lethal interaction motifs and a pathway identification algorithm based on synthetic lethal interaction datasets, the model holds good potential as an integrative method which combines multiple sources of evidence. If the sources of evidence are independent, the new likelihood function should be the multiplication of those for individual evidences. When the sources of evidence are not independent, then a Bayesian learning approach such as the framework developed by Jansen et al. [29] should be considered. A detailed discussion on the extension of GIMF into an integrative approach is however, beyond the scope of this study and hence will not be further considered here.

## Conclusion

A probabilistic model and an automated algorithm (GIMF) have been shown to be effective in unsupervised motif learning of genetic interaction data. Starting from a seed pattern of genetic interaction partners, the method iteratively identifies genes that share the pattern and characterizes the pattern with a probabilistic motif. Functional associations are inferred from motif membership, rather than from existence of a direct genetic interaction linking two genes. Genes that belong to the same connected components in Type I and Type II networks have well correlated GO annotations, and are more likely to share annotations than genes connected by direct synthetic lethal interactions. Synthetic lethal interactions tend to be depleted between genes within a motif, suggesting that synthetic lethal interactions occur primarily between-pathway rather than within-pathway.

Several desirable features of the proposed algorithm for analyzing genetic interaction data include strong 0/1 predictions for genes sharing a motif, asymmetric property and the ability to automatically down-weight the impact of promiscuous genes with large degrees. We have shown that the asymmetry can be exploited to identify even tighter associations between genes and mask the impact of promiscuous genes. Furthermore, we conjecture that this asymmetric property may be useful in discriminating genes that are exclusive to a single pathway from genes that are shared in multiple pathways.

The probabilistic motifs naturally down-weight the importance of promiscuous genes with many interaction partners. When the roles of hubs are not purely due to experimental bias, it is more likely to retain biologically relevant information by modelling it probabilistically than by simple filtering. GIMF has an interesting correspondence with a log-odd score based approach. However, an important difference is GIMF performs a global search of a subgraph with best cohesiveness based on a seed. The computation of GIMF is highly efficient. It is well suited for building motifs around a subset of genes of interest with several choices of stringency.

## Methods

Correlations for Gene Ontology (GO) annotation are computed for three categories: biological process, molecular function and cellular component (unpublished data, Ye et al). Within each category, the correlation coefficient is computed as follows:

Find the deepest level in GO hierarchy at which the pair of genes shares an annotation, which we denote by *d*.

Find the maximum and minimum value of *d *among all query gene pairs (*i, j*) where *i *= 1,2,..., *Q *and *j *= 1,2,...,*Q*, *Q *is the total number of query genes.

The GO annotation correlation (biological process, molecular function and cellular components) for a pair of gene is defined by

correlation=d−dmin⁡dmax⁡−dmin⁡     (12)
 MathType@MTEF@5@5@+=feaafiart1ev1aqatCvAUfKttLearuWrP9MDH5MBPbIqV92AaeXatLxBI9gBaebbnrfifHhDYfgasaacH8akY=wiFfYdH8Gipec8Eeeu0xXdbba9frFj0=OqFfea0dXdd9vqai=hGuQ8kuc9pgc9s8qqaq=dirpe0xb9q8qiLsFr0=vr0=vr0dc8meaabaqaciGacaGaaeqabaqabeGadaaakeaacqWGJbWycqWGVbWBcqWGYbGCcqWGYbGCcqWGLbqzcqWGSbaBcqWGHbqycqWG0baDcqWGPbqAcqWGVbWBcqWGUbGBcqGH9aqpdaWcaaqaaiabdsgaKjabgkHiTiabdsgaKnaaBaaaleaacyGGTbqBcqGGPbqAcqGGUbGBaeqaaaGcbaGaemizaq2aaSbaaSqaaiGbc2gaTjabcggaHjabcIha4bqabaGccqGHsislcqWGKbazdaWgaaWcbaGagiyBa0MaeiyAaKMaeiOBa4gabeaaaaGccaWLjaGaaCzcamaabmaabaGaeGymaeJaeGOmaidacaGLOaGaayzkaaaaaa@55C5@

## Authors' contributions

YQ developed the GIMF model and carried out the data analysis. PY provided the code for the calculation of GO correlations and suggested the test of robustness against hub removal. JSB supervised the study.

## Supplementary Material

Additional File 1 Supplementary methods, figures, and tables. Click here for file

Additional File 2 Complete list of motif members for the 126 query genes using initialization *p = 0.95*. Click here for file

Additional File 3 Tar file containing Matlab code, input, and output. Click here for file
