# Supplementary material for: Genetic interaction motif finding by expectation maximization – a novel statistical model for inferring gene modules from synthetic lethality
Source: BMC Bioinformatics. 2005 Dec 6;6:288. doi: 10.1186/1471-2105-6-288 (PMC1334220; doi:10.1186/1471-2105-6-288)
Supplement: Additional File 1 — Supplementary methods, figures, and tables. [file 1471-2105-6-288-S1.pdf]

Supplementary material

Fig. S1 False positives of GIMF on randomized datasets

Distributions of (a) the number of seeds that lead to false positives and (b) number of false positives by running GIMF on 100 randomized networks. The histograms are fit with Poisson distributions.

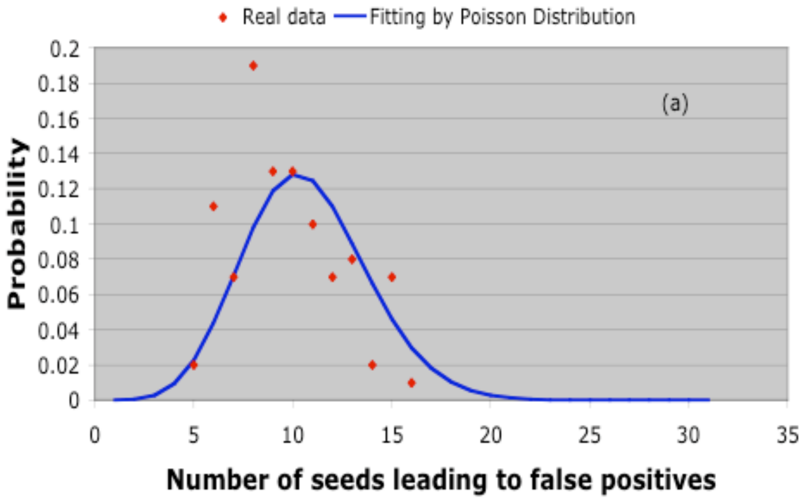

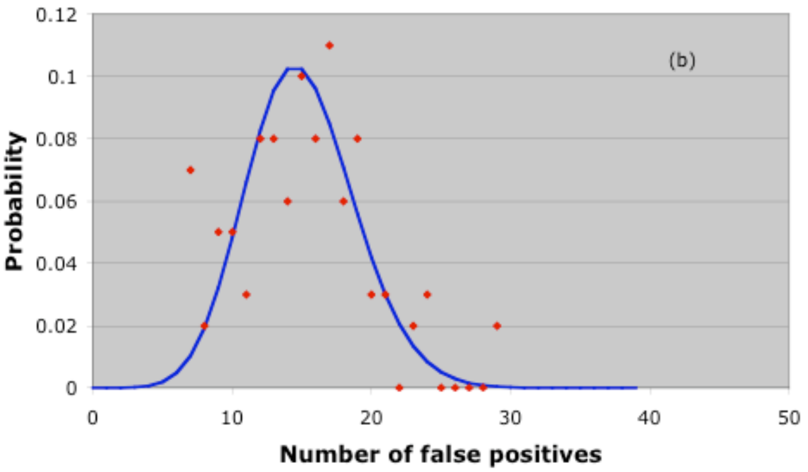

Fig. S2. Distribution of distinct motifs

Motifs identified by EM represent local maxima of a likelihood function. These local maxima are visualized by projecting the motifs into a 2-dimensional space using classical multidimensional scaling using a distance matrix defined by the Euclidean distance between the probability vectors defining each motif. This projection is essentially equivalent to principal component analysis.

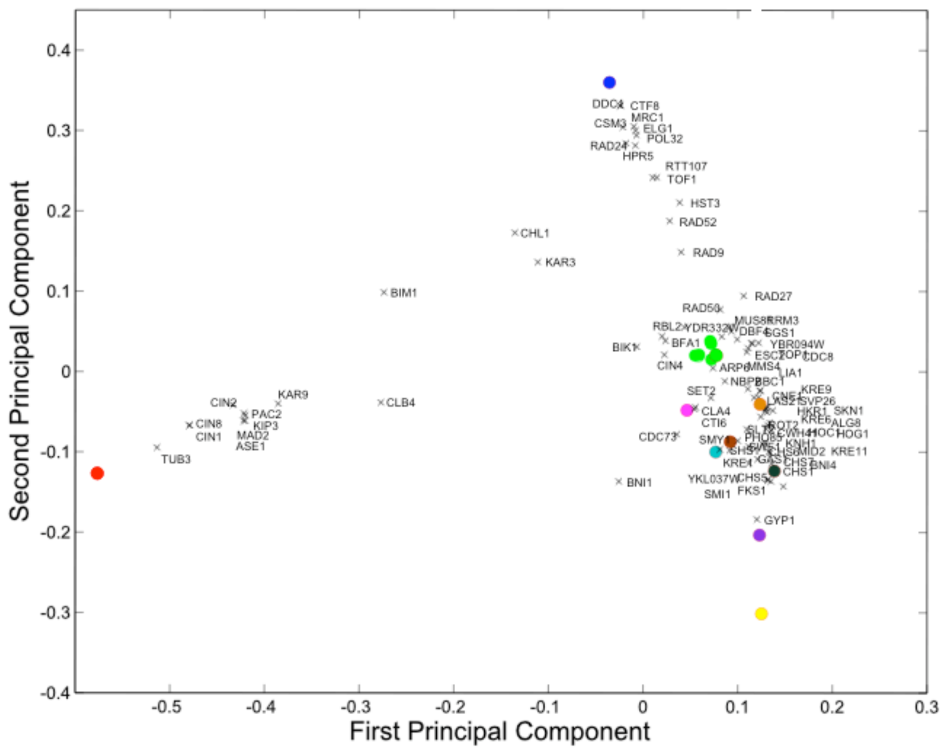

Fig. S3. Degree distribution of query genes

Query genes are binned according to the number of interaction partners; bins are labeled by midpoint values. The number of confirmed interactions per query gene varies from 1 to 146, with an average of 34 interactions. The tail of the histogram indicates the existence of hub genes. For example, 9 out of 126 query genes have more than 100 (>3 standard deviation) interaction partners.

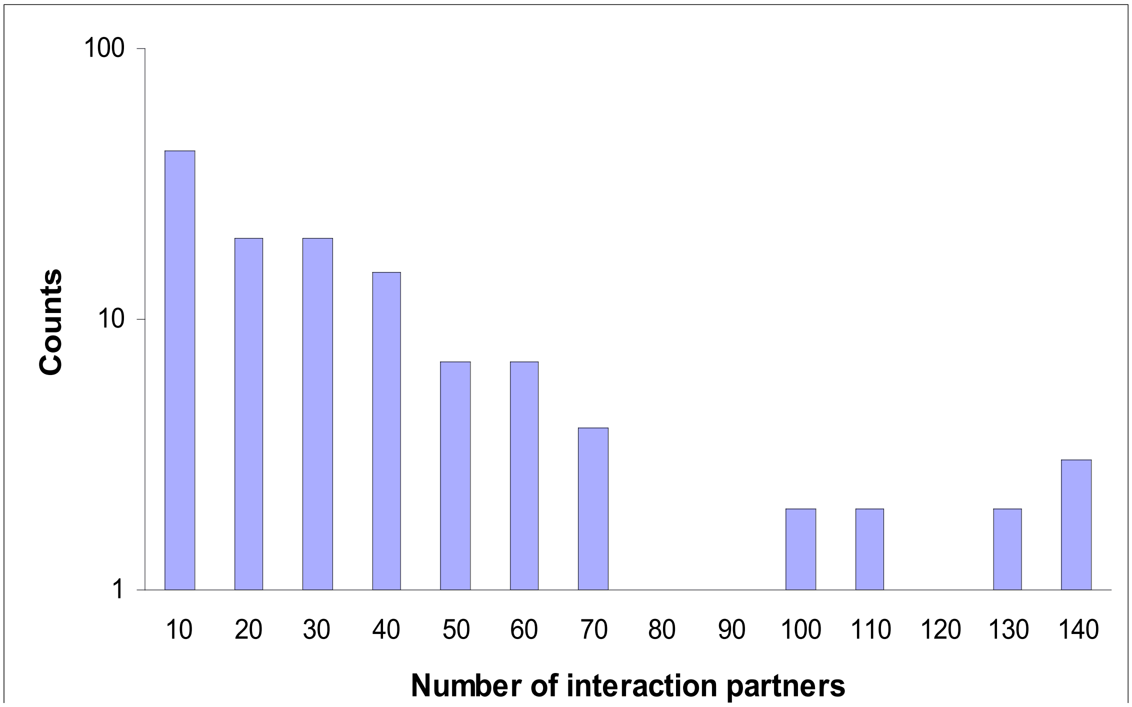

Fig. S4. Degree distribution of library genes

Library genes are binned according to the number of interaction partners; bins are labeled by midpoint values. The number of confirmed interactions per library gene varies from 1 to 43, with an average of 4.3 interactions. There are 35 out of 982 library genes that have more than 16 interactions (> 3 standard deviation).

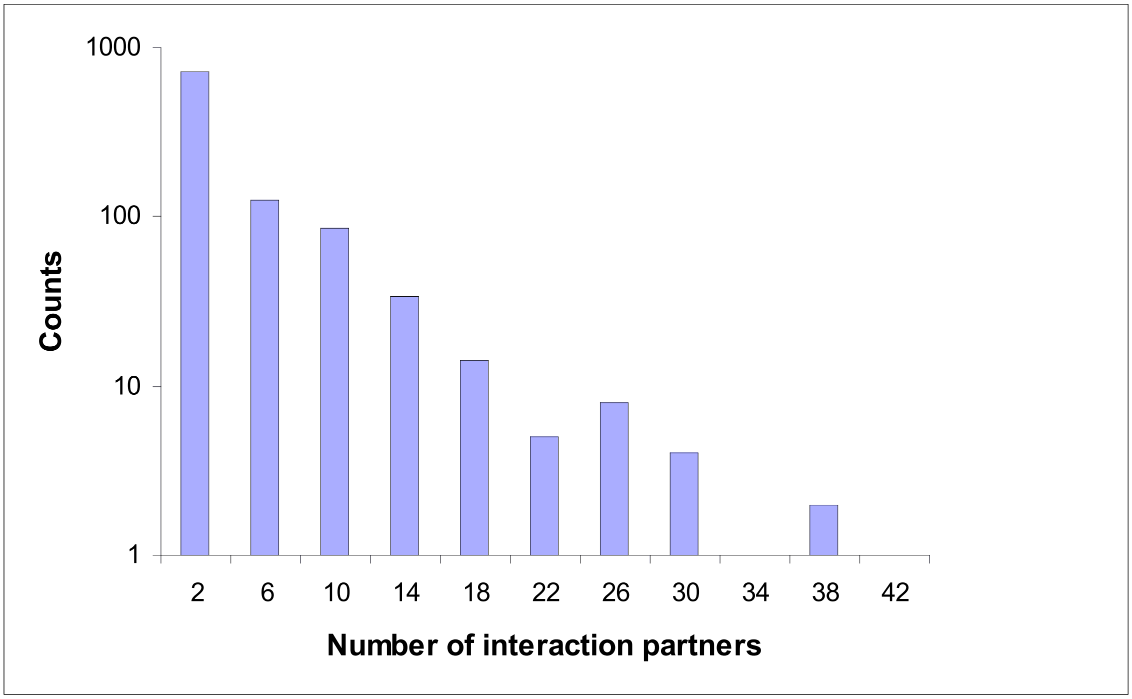

Fig. S5. Visualization of the synthetic lethal interaction network.
Query genes are red; library genes are green; and genes with interactions as both query and library are yellow. The size of a node indicates its number of interaction partners.

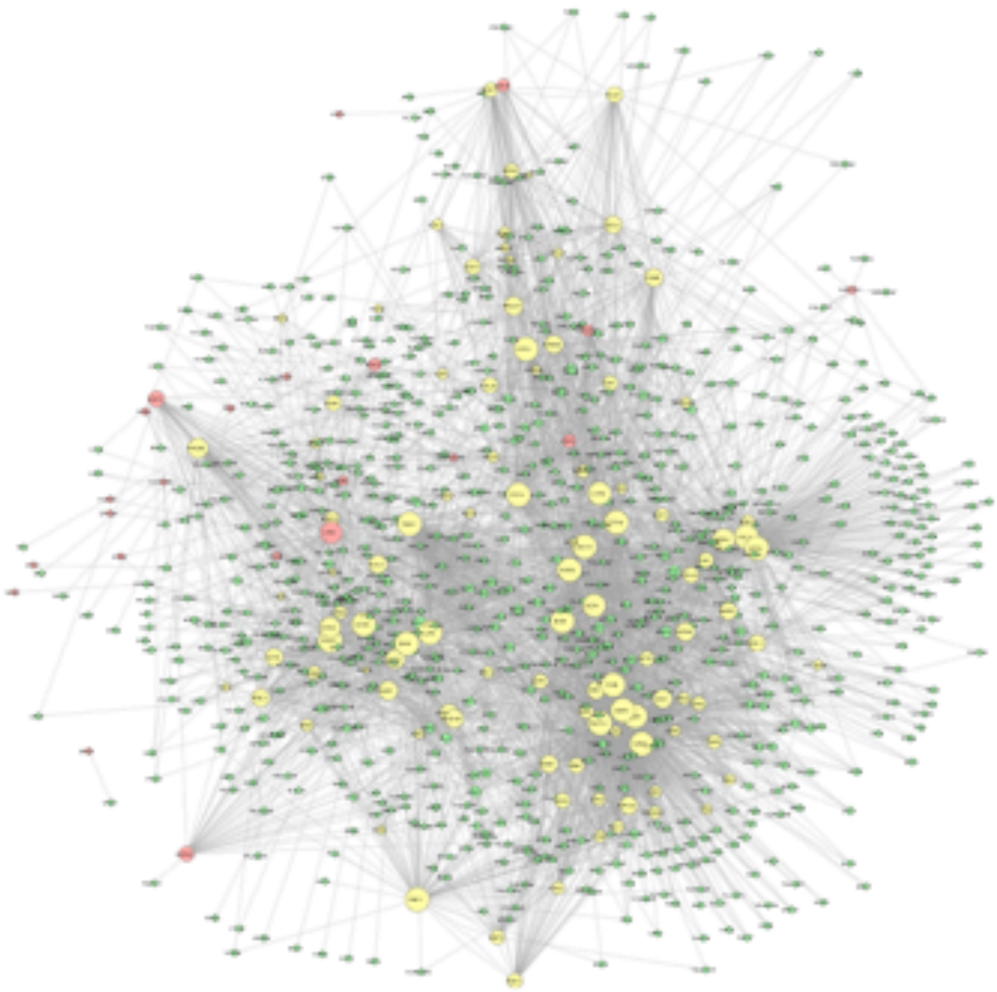

Table S1. Classification of query seed genes based on stability of their motif members.

Denote the set of motif members for seed gene i obtained with initialization parameter p by M_p^i. The corresponding Jaccard coefficient J_p^i is given by $J_p^i = \frac{M_p^i \cap M_{p=0.95}^i}{M_p^i \cup M_{p=0.95}^i}$. The query genes are classified into five categories according to the Jaccard coefficient averaged over four different values of p: (1) "S*" (very strong) if $\bar{J}^i = 1$; (2) "S" (strong) if $0.9 < \bar{J}^i < 1$; (3) "MS": (moderately strong) if $0.6 < \bar{J}^i < 0.9$; (4) "W": (weak) if $\bar{J}^i < 0.6$; (5) "NA": (not available) if the query gene has less than 5 interactions and hence is not used as a seed.

Gene ID	ORF name	Jaccard coefficient				Category
		p=0.6	p=0.7	p=0.8	p=0.9	
1	YAL013W	0.67	1.00	1.00	1.00	S
2	YAL058W	0.10	0.11	0.18	0.33	W
3	YBL001C			NA		
4	YBL061C	0.60	0.75	0.75	0.75	MS
5	YBR023C	0.60	0.75	1.00	1.00	MS
6	YBR094W	0.13	0.25	0.33	0.33	W
7	YBR098W	0.38	0.39	0.42	0.73	W
8	YBR164C	0.80	1.00	1.00	1.00	S
9	YBR205W			NA		
10	YBR229C	0.20	0.33	0.33	0.33	W
11	YBR234C	0.18	0.40	0.50	1.00	W
12	YCL016C	0.75	0.75	0.75	0.75	MS
13	YCL029C	0.13	0.14	0.14	0.14	W
14	YCL051W			NA		
15	YCL061C	0.36	0.36	0.36	0.50	W
16	YCR009C	0.50	0.50	1.00	1.00	MS
17	YDL029W	0.33	0.50	1.00	1.00	MS
18	YDL049C	1.00	1.00	1.00	1.00	S*
19	YDL225W	0.44	0.50	0.53	1.00	MS
20	YDR052C	1.00	1.00	1.00	1.00	S*
21	YDR126W	0.25	0.33	0.33	1.00	W
22	YDR150W	0.09	0.13	0.25	0.67	W
23	YDR162C	0.10	0.11	0.13	1.00	W
24	YDR217C	0.26	0.29	0.33	0.50	W
25	YDR332W	0.08	0.08	0.17	0.33	W
26	YDR363W	0.50	1.00	1.00	1.00	MS
27	YDR386W	0.55	0.62	0.64	0.80	MS
28	YDR388W	0.50	0.50	1.00	1.00	MS
29	YDR420W	0.17	0.25	0.25	0.50	W
30	YDR424C	0.55	0.60	0.67	0.80	MS
31	YDR488C	0.50	0.52	0.56	0.85	MS
32	YEL003W	0.71	0.83	1.00	1.00	MS
33	YEL061C	0.78	0.78	0.88	0.88	MS
34	YER007W	0.67	0.67	0.73	0.80	MS

35	YER016W	0.10	0.14	0.17	0.33	W
36	YER173W	0.25	0.31	0.33	0.63	W
37	YGL027C	0.20	0.25	0.25	0.33	W
38	YGL216W	0.62	0.62	0.62	0.89	MS
39	YGR032W			NA		
40	YGR078C	1.00	1.00	1.00	1.00	S*
41	YGR143W	0.50	0.67	1.00	1.00	MS
42	YGR166W	0.33	0.33	0.50	0.50	W
43	YGR227W	0.19	0.33	0.75	0.75	W
44	YGR229C	1.00	1.00	1.00	1.00	S*
45	YGR284C			NA		
46	YHR030C	0.33	0.50	1.00	1.00	MS
47	YHR031C	0.09	0.10	0.14	0.33	W
48	YHR129C	0.53	0.73	0.80	0.89	MS
49	YHR142W	0.50	0.67	1.00	1.00	MS
50	YHR154W	0.20	0.33	0.67	1.00	W
51	YHR181W	0.11	0.20	0.50	1.00	W
52	YHR191C	0.57	0.80	1.00	1.00	MS
53	YHR204W			NA		
54	YJL020C	0.20	0.20	0.25	1.00	W
55	YJL030W	0.80	0.80	0.89	1.00	MS
56	YJL062W	1.00	1.00	1.00	1.00	S*
57	YJL092W	0.11	0.14	0.25	0.67	W
58	YJL099W	0.50	0.60	0.60	0.60	W
59	YJL139C			NA		
60	YJL168C	0.09	0.20	0.33	1.00	W
61	YJL174W	1.00	1.00	1.00	1.00	S*
62	YJR043C	0.27	0.27	0.30	0.43	W
63	YJR053W	0.34	0.49	0.49	0.68	W
64	YJR057W	1.00	1.00	1.00	1.00	S*
65	YJR070C	0.25	1.00	1.00	1.00	MS
66	YJR075W	0.33	1.00	1.00	1.00	MS
67	YJR131W			NA		
68	YKL037W	0.30	0.50	0.75	1.00	MS
69	YKL079W	0.82	1.00	1.00	1.00	S
70	YKL113C	0.10	0.13	1.00	1.00	W
71	YKR054C	0.56	0.64	0.64	1.00	MS
72	YLR039C	1.00	1.00	1.00	1.00	S*
73	YLR057W			NA		
74	YLR085C	0.50	0.50	1.00	1.00	MS
75	YLR113W	1.00	1.00	1.00	1.00	S*
76	YLR200W	0.83	1.00	1.00	1.00	S
77	YLR210W	1.00	1.00	1.00	1.00	S*
78	YLR262C	1.00	1.00	1.00	1.00	S*
79	YLR286C			NA		
80	YLR330W	0.33	0.50	0.50	1.00	W
81	YLR332W	0.42	0.56	0.63	1.00	MS
82	YLR342W	0.50	0.50	0.50	0.50	W
83	YLR418C	1.00	1.00	1.00	1.00	S*

84	YML032C	0.19	0.20	0.43	0.75	W
85	YML094W	0.83	1.00	1.00	1.00	S
86	YML124C	0.78	0.88	0.88	0.88	MS
87	YMR048W	0.29	0.33	0.40	1.00	W
88	YMR078C	0.43	0.60	0.75	0.75	MS
89	YMR138W	0.59	0.61	0.61	1.00	MS
90	YMR190C	0.13	0.25	0.33	0.50	W
91	YMR263W	0.40	0.67	0.67	1.00	MS
92	YMR294W	0.53	0.62	0.67	1.00	MS
93	YMR299C	0.39	0.40	0.67	0.86	W
94	YMR307W	0.33	0.33	1.00	1.00	MS
95	YNL098C			NA		
96	YNL153C	0.71	0.83	0.83	1.00	MS
97	YNL192W	1.00	1.00	1.00	1.00	S*
98	YNL233W	0.20	0.20	0.25	0.50	W
99	YNL250W	0.50	0.50	0.50	0.50	W
100	YNL271C	0.14	0.50	0.50	0.50	W
101	YNL273W	0.58	0.70	0.78	1.00	MS
102	YNL298W	0.33	1.00	1.00	1.00	MS
103	YNL322C	0.50	1.00	1.00	1.00	MS
104	YOL006C	0.11	0.33	0.33	1.00	W
105	YOL068C			NA		
106	YOR002W	0.30	0.43	0.75	1.00	MS
107	YOR025W	0.21	0.21	0.23	0.38	W
108	YOR058C	0.62	0.62	0.89	1.00	MS
109	YOR067C	0.08	0.08	0.20	1.00	W
110	YOR070C	0.33	0.33	0.33	0.67	W
111	YOR144C	0.27	0.30	0.50	0.75	W
112	YOR265W	0.41	0.41	0.38	0.48	W
113	YOR269W	0.53	0.75	0.90	1.00	MS
114	YOR349W	0.80	0.80	0.80	0.89	MS
115	YPL008W	0.36	0.50	0.50	0.50	W
116	YPL031C	1.00	1.00	1.00	1.00	S*
117	YPL051W	0.67	0.67	1.00	1.00	MS
118	YPL155C	0.26	0.23	0.25	0.28	W
119	YPL174C	0.25	0.30	0.38	0.43	W
120	YPL181W	0.30	0.30	0.30	0.33	W
121	YPL194W	0.38	0.63	0.83	1.00	MS
122	YPL241C	0.82	0.90	0.90	1.00	S
123	YPL269W	0.69	0.69	0.69	1.00	MS
124	YPR135W	0.17	0.25	0.25	1.00	W
125	YPR141C	0.11	0.11	0.11	0.33	W
126	YPR159W	0.50	1.00	1.00	1.00	MS

Table S2: Information on eight seed genes that are not part of their motifs

Seed	No. of interactions	No. of motif genes	Average No. of interactions for a motif gene
YDL225W	6	7	57
YER007W	11	8	108
YKL037W	6	2	73
YLR210W	10	13	81
YML124C	21	6	120
YOR349W	31	7	118
YPL194W	14	4	68
YPL241C	18	8	112
